# DCTN2 inhibited cellular senescence through targeting TRIM21 mediated MST2 ubiquitination and activation in hepatocellular carcinoma

**DOI:** 10.7150/thno.137790

**Published:** 2026-09-03

**Authors:** Yanbo Wang, Yaomei He, Qin Yu, Ningrui Wang, Qiaoling Ruan, Haiyan Wang, Hanqing Zhao, Yonglin Zhang, Yang Ke, Caie Tang, Liqi Lang, Yuxia Tang, Lixia Liu, Hao Zhang, Shan Yan, Aimin Wang, Qiushuo Shen, Ceshi Chen

**Affiliations:** 1 Department of Geriatric Medicine, The Affiliated Hospital of Qingdao University, Qingdao University, Qingdao, 266000, China.; 2 Yunnan Key Laboratory of Breast Cancer Precision Medicine, Institute of Biomedical Engineering, Kunming Medical University, Kunming, 650500, Yunnan, China.; 3 Yunnan Key Laboratory of Breast Cancer Precision Medicine, Yunnan Cancer Hospital, The Third Affiliated Hospital of Kunming Medical University, Yunnan Hospital of Peking University Cancer Hospital, Kunming, 650118, Yunnan, China.; 4 Center for Life Sciences, Yunnan Key Laboratory of Cell Metabolism and Diseases, School of Life Sciences, Yunnan University, Kunming 650500, China.; 5 School of Nursing, Qingdao University, Qingdao 266021, China.; 6 School of Pharmacy, Qingdao University, Qingdao 266021, China.; 7 Institute of Basic Medicine and Forensic Medicine, North Sichuan Medical College, Nanchong, 637100, China.; 8 The Department of Hepatobiliary Surgery, The Second Affiliated Hospital of Kunming Medical University, Kunming, 650101, China.

**Keywords:** hepatocellular carcinoma, cellular senescence, dynactin subunit 2, Hippo signaling pathway, tripartite motif-containing 21

## Abstract

**Rationale:**

Hepatocellular carcinoma (HCC) is a leading cause of cancer-related mortality, and inducing cellular senescence presents a promising therapeutic approach. However, the mechanisms underlying senescence in HCC pathogenesis remain unclear. This study investigated key genes involved in liver cancer senescence.

**Methods:**

Bioinformatic analyses of HCC datasets identified senescence-related genes, highlighting dynactin subunit 2 (*DCTN2*). We investigated DCTN2’s biological function and regulatory mechanism, analyzed its association with patient prognosis, evaluated the synergistic effects of vilazodone and sorafenib *in vitro* and *in vivo*, and examined tumor protein p53 (*TP53*) mutation-mediated regulation of DCTN2.

**Results:**

DCTN2 was identified as a novel HCC senescence-associated gene, whose expression was transcriptionally upregulated by TP53 mutation. DCTN2 knockdown induced cellular senescence, thereby inhibiting HCC cell proliferation, migration, invasion and angiogenesis, whereas its overexpression exerted an opposite oncogenic effect. DCTN2 repressed cellular senescence via interaction with TRIM21, which decreased MST2 K63-linked polyubiquitination and ultimately attenuated the Hippo signaling pathway; *DCTN2* overexpression correlated with poor HCC prognosis. Moreover, targeting TRIM21 with vilazodone and sorafenib synergistically induced HCC cellular senescence *in vitro and in vivo*.

**Conclusions:**

Our findings revealed that DCTN2 suppresses cellular senescence by inhibiting the Hippo signaling pathway in HCC. Targeting the DCTN2/TRIM21/MST2 axis, along with TP53 mutation regulation, offers new therapeutic insights for HCC treatment.

## Introduction

Hepatocellular carcinoma (HCC) represents a major health challenge, accounting for roughly 90% of all liver cancer cases and ranking as the third leading cause of cancer-associated deaths worldwide 1, 2. Despite advances in early detection, surgical interventions and targeted therapies (e.g., sorafenib and lenvatinib), the prognosis for patients diagnosed with HCC remained poor, especially in the context of advanced-stage disease and metastasis 3. Therefore, it is crucial to decipher the deeper molecular mechanisms driving HCC progression, and develop novel diagnostic biomarkers and therapeutic strategies.

Cellular senescence manifests as irreversible cell cycle arrest accompanied by morphological and macromolecular alterations. Induced by diverse endogenous and exogenous stressors (e.g., mitochondrial dysfunction, DNA damage, oncogene activation), it is recognized as an emerging cancer hallmark 4, 5. Senescent cells exhibited reduced proliferative potential, elevated senescence-associated β-galactosidase (SA-β-gal) activity, and upregulation of cell-cycle inhibitory proteins such as p16 and p21 6, 7. Moreover, senescent cells produce various bioactive molecules that constitute the senescence-associated secretory phenotype (SASP), such as cytokines, chemokines and growth factors 8. Through sophisticated intercellular communication networks, cellular senescence orchestrates the pro-inflammatory and anti-inflammatory microenvironment, dynamically modulating tissue homeostasis and liver disease across various stages including HCC 9. Consequently, cellular senescence acts as a “double agent” in HCC progression. Tumor cellular senescence induces cell cycle arrest and triggers immunosurveillance to eradicate senescent malignant cells and their neighboring non-senescent pre-malignant cells, thereby impeding tumor development 10. Conversely, senescent tumor cells not only contribute to the genomic instability and enhance invasiveness of HCC but also remodel tumor microenvironment to promote tumor stemness, metastasis and immune escape through SASP 11, 12. Nevertheless, the induction of cellular senescence provides a promising strategy for HCC combination therapies.

The Hippo signaling pathway is evolutionarily conserved and modulates various cellular processes including cell survival, proliferation, differentiation, tumorigenesis and organ size 13. In vertebrates, the Hippo signaling pathway comprises several core components, such as the serine/threonine kinases MST1/2 (homologs of Hippo, also known as STK4 and STK3), large tumor suppressor kinase 1/2 (LATS1/2), and Yes-associated protein (YAP)/ transcriptional coactivator with PDZ binding motif (TAZ, a paralog of YAP in mammals) 14. Normally, upstream stimuli induce dimerization and autophosphorylation of MST and activate LATS1/2, and then phosphorylated LATS1/2 further phosphorylate YAP/TAZ, leading to the cytosolic retention and proteasome-mediated degradation of YAP/TAZ, which represses the interaction of YAP/TAZ with transcription factors and negatively regulates downstream gene expression 13, 15. Moreover, the Hippo pathway plays important roles in regulating tumor cellular senescence. For instance, YAP promotes cell proliferation and inhibits cellular senescence by transcriptionally up-regulating cyclin D-dependent kinase 6 (*CDK6*) gene expression 16.

In this study, we identified dynactin subunit 2 (*DCTN2*) as a new cellular senescence-associated gene and demonstrated that DCTN2 provoked cellular senescence through targeting TRIM21-mediated MST2 activation in HCC.

## Methods

### Cell culture and cell lines establishment

The cell lines utilized in this study were obtained from the American Type Culture Collection (ATCC) with STR certificates and cultured under standard conditions using culture medium recommended by ATCC. LO2, SNU-182, Huh-7, HEK-293T, and MHCC97H cells were maintained in DMEM containing 10% fetal bovine serum (FBS) plus 1% penicillin/streptomycin (Gibco, Cat#11995065), HepG2, Hep3B, and SK-hep-1 cells were cultured in MEM containing 10% FBS and 1% penicillin/streptomycin (Gibco, Cat#11095080), while THLE-2 cells were cultured in BEBM containing 10% FBS and 1% penicillin/streptomycin (ZQXZ-bio, Cat#ZQ-1340). HepG2-TP53-KO cell lines were gifts from Professor Lianghu Qu (Sun Yat-sen University). Human umbilical vein endothelial cells (HUVECs) were cultured in the human large vessel endothelial cell-specific basal medium (Gibco, Cat#M200PRF500). All cells were grown in a 37 °C incubator with 5% CO_2_ in a humidified environment. For the overexpression or knockdown of DCTN2 in HCC cells, the full-length human DCTN2 cDNA was synthesized by Shanghai Generay Biotech and subcloned into the pCDH-CMV-E2F-eGFP lentiviral vector. For DCTN2 knockdown, two independent shRNA targeting different regions of the DCTN2 mRNA were constructed using the pLKO.1 vector (Addgene). Lentivirus was generated according to the manufacturer’s protocol. Briefly, viral supernatants containing different lentiviruses were collected from HEK-293T cells 48 h and 72 h after transfection and used to infect target HCC cells. Positive infected cells were selected using puromycin. Additionally, synthesized siRNA (from Shanghai Sangon Biotechnology) was transfected into target tumor cells using the Lipofectamine RNAiMAX (Invitrogen, Cat#13778) reagent. The sequences of shRNA and siRNA were included in Table S1.

### Cell growth, BrdU staining and Colony formation assays

12-well plates were seeded with MHCC97H and SK-hep-1 cells separately at 4×10^4^ or 2×10^4^ cells per well. Cell densities were quantified daily using an automated cell counter (Countstar, Shanghai Ruiyu Biotech Co) to generate growth curves. For BrdU staining assay, cells were incubated for 24 h prior to treatment with 10 μM BrdU (Thermo, Cat#B23151), followed by fixation in 4% paraformaldehyde (PFA). Samples were incubated overnight with the primary anti-BrdU antibody (Cell Signaling Technology, Cat#5292S), and secondary antibody detection was performed (Thermo, Cat#A11005). Nuclei were stained with 4’,6-diamidino-2-phenylindole (DAPI). Cells were trypsin digested, resuspended and counted, and then seeded into 6-well plates at 300-800 cells per well in 2 mL cell culture medium supplemented with 10% FBS for colony formation assay. Medium was refreshed every three days for a total culture duration of 2–3 weeks. After fixation with 4% PFA, colonies were stained with crystal violet and counted.

### Wound-healing and trans-well assays

To assess the impact of DCTN2 on the migration of HCC cells, wound-healing and trans-well migration assays were conducted as previously described 17. Briefly, after cells reached confluence in 6-well plates, monolayers were scratched with sterile pipette tips to form wounds. PBS was applied to remove any detached cells. The wound area was imaged at specified time intervals and analyzed using ImageJ software. For the trans-well migration assay, 2.5×10^4^ cells were seeded in the upper chamber of a 24-well plate insert (Corning Life Sciences), with the lower chamber filled with medium containing 10% FBS. The cells were allowed to migrate for the indicated hours. After wiping non-migrating cells off the upper chamber, migrated cells were fixed with 4% PFA, followed by crystal violet staining and cell counting. For the invasion assay, matrigel (Corning, Cat#356234) diluted 1:9 with serum-free medium was coated on the upper chamber of a 24-well plate insert (Corning Life Sciences). 2×10^5^ cells were seeded into the coated upper chamber, and medium supplemented with 10% FBS was added to the lower chamber. The cells were allowed to invade for indicated hours, with subsequent steps following the standard trans-well protocol.

### Flow cytometry analysis and real-time quantitative PCR (RT-qPCR)

To examine the impact of DCTN2 knockdown on cell cycle distribution and apoptosis, flow cytometry analysis was performed. Cells were starved for 12 h, then cultured in complete medium for another 12 h. After washing with cold PBS, they were harvested, fixed in 70% ethanol overnight at 4 °C, and stained with propidium iodide (PI) at 37 °C for 1 h. To detect cellular apoptosis, cells were collected with EDTA-free trypsin, washed twice using cold PBS, resuspended in 1× binding buffer, and stained with Annexin V-FITC and PI at room temperature under dark conditions for 15 min. All samples were analyzed on the Sony ID7000 Flow Cytometer. RT-qPCR was adopted to detect the expression levels of target genes. Total RNA was isolated with Invitrogen TRIzol reagent (Thermo, Cat#15596018) following the standard protocol and subsequently reverse transcribed into cDNA using a reverse transcription kit (Vazyme, Cat#R333-01). To evaluate gene expression, cDNA was analyzed by RT-qPCR with FastStart Universal SYBR Green Master Mix (Vazyme, Cat#Q712-02). β-actin was used as the internal control. All RT-qPCR primer sequences were listed in Table S1.

### Western blot

Proteins were analyzed by performing a BCA Protein Quantification Assay (Abbkine, Cat#KTD3001) to assess the protein concentration in cell and tissue samples that were lysed with RIPA buffer (Beyotime, Cat#P0013B). Proteins were separated by SDS-PAGE and transferred onto a PVDF membrane. The membrane was then blocked with 5% non-fat milk in TBS buffer containing 0.1% Tween-20 (TBST) for 2 h at room temperature. Subsequently, the membrane was incubated overnight with specific primary antibodies. Primary antibody details are listed in Table S1, and full-length gel/blot images are included in Data S3. The secondary antibodies conjugated with horseradish peroxidase (HRP) goat anti-mouse IgG (Thermo, Cat#31430) or goat anti-rabbit IgG (Thermo, Cat#31460) were utilized and detected using MiniChemi imaging equipment for visualization.

### Animal experiments

Five-week-old male BALB/c nude mice, sourced from Beijing Vital River Laboratory Animal Technology Co., Ltd., with all experimental protocols preapproved and conducted in line with the Animal Care and Use Committee of Kunming Medical University (Ethics no. KMMUX202505072), were randomly assigned to various experimental groups, six mice per group (n = 6). Xenograft models were created by subcutaneously injecting 8×10^6^ SK-hep-1 cells into the BALB/c nude mice. Tumor growth was monitored and measured every seven days, with the tumor sizes recorded. Tumor volumes were calculated using the formula (L × W^2^)/2, where L represented the tumor length and W represented the tumor width. After six weeks of implantation, the mice were euthanized, and the xenografts were surgically removed. In the *in vivo* drug treatment experiment, designated cells were subcutaneously injected into mice. When the xenograft tumor volume reached 50 mm^3^, mice were intraperitoneally injected with vilazodone (25 mg/kg), sorafenib (10 mg/kg), or a combination of these two drugs, which were administered every two days for 3 weeks. The tumors were then weighed and processed for immunohistochemistry (IHC) with specific antibodies.

### Hematoxylin-eosin staining (H&E) and IHC

Xenograft tumors specimens were collected, fixed with formaldehyde for two days, embedded in paraffin, and subsequently sectioned. H&E staining of these sections was performed for pathological analysis as previously reported 17. IHC was performed following standard procedures. Briefly, tissue sections were treated with xylene, rehydrated, and antigen retrieval was done with citrate solution. After blocking with 10% normal goat serum, sections were incubated with primary antibodies (Table S1), washed with PBS three times for 2 min each; sections were incubated with HRP polymer (Reagent C, KeyGEN, Cat#KGC3201-300) for 30 min under ambient temperature, then washed twice in PBS, 2 min per wash. DAB chromogenic solution (Reagents D/E/F, KeyGEN, Cat#KGC3201-300) was added dropwise, and developed at room temperature for 2–5 min, and stained with hematoxylin. IHC images were captured using a Nikon ECLIPSE microscope. DCTN2 expression in clinical samples was validated using a tissue microarray as previously described 16 of paired 90 HCC cases from Shanghai Outdo Biotech Co., Ltd (HLivH180Su09), which was conducted with the approval of the Institutional Scientific Research Ethics Committee at the Kunming Medical University (Ethics no. KMMU2024MEC189). Two blinded pathologists independently scored immunohistochemical staining as reported 18. Briefly, staining intensity was scored as 0 (negative), 1 (weak), 2 (moderate), and 3 (strong). The proportion of positive tumor cells was scored as 0 (0%), 1 (1–25%), 2 (26–50%), 3 (51–75%), and 4 (76–100%). The final immunoreactivity score was calculated by the product of the intensity and proportion scores. Cases were divided into low- and high-expression groups using a cutoff value of ≥ 8 for high expression and < 8 for low expression. Full clinicopathologic characteristics of the tissue microarrays (TMA) cohort and logistic regression analyses are provided in Table S3 and Data S2.

### SA-β-gal staining assay

For SA-β-gal staining, the indicated cells were plated into 96-well plates. After overnight incubation, the medium was removed, and cells were washed thrice with PBS prior to fixation and staining with the Cell Senescence β-Galactosidase Staining Kit (Beyotime, Cat#C0602). Stained cells were observed under a microscope, and the percentage of senescent cells was quantified by calculating the number of SA-β-Gal-positive cells in randomly selected areas (n = 5).

### In vitro HUVEC tube formation assay

Logarithmically growing DCTN2 knockdown and overexpression cells were trypsinized, dispensed into 6-well plates at 5×10^5^ cells/well, and cultured at 37 ℃ with 5% CO_2_ for 24 h to 60-80% confluency. After two washes with sterile PBS, 2 mL serum-free DMEM was added for another 24 h of culture. Supernatants (conditioned medium, CM) were collected, centrifuged at 3,000 rpm for 10 min at 4 ℃, filtered through a 0.22 μm membrane, aliquoted, and stored. Prior to the assay, 96-well plates and pipette tips were pre-cooled at -20 ℃ for 2 h. On ice, 50 μL Matrigel (Corning, Cat#354230) per well was added to pre-cooled plates, which were solidified at room temperature for 1 h or 37 ℃ for 30 min. HUVECs were deprived of serum for 3-6 h, trypsinized, centrifuged, and resuspended in CM supplemented with 1% FBS. The cells were seeded onto Matrigel-coated plates with ≥3 replicate wells per group, incubated at 37 ℃ under 5% CO_2_ for 4-6 h, after incubation, the cells were stained with Calcein-AM working solution (Beyotime, Cat#C2012), and tubular structures were imaged under a microscope for subsequent quantitative analysis.

### Luciferase assay

24-well plates were seeded with HEK-293T cells. Transfection was performed using target plasmids and pGL3 luciferase reporter plasmids (300 ng/well), plus renilla control plasmid (10 ng/well). 48 h post-transfection, cell lysates were collected, and luciferase activity was detected using the Dual-Luciferase Reporter Assay System following the standard protocol (Beyotime, Cat#RG027). The primer information for plasmid construction was listed in Table S1 of the supporting information.

### Immunofluorescence staining

Cells were seeded into 8-well plates at a density of 1-2×10⁴ cells/well. On the next day, cells were fixed with 4% PFA for 15 min, followed by three washes with PBS. For permeabilization, 500 μL permeabilization buffer (PBS containing 0.2% Triton X-100) was added to each well, and cells were incubated for 10 min. Cells were then blocked with 10% NGS for 2 h, kept in primary antibody solution overnight at 4 °C, followed by three PBS washes. Subsequent incubation with secondary antibody proceeded over 2 h, followed by three additional PBS washes. Positive signals were observed under a Confocal Laser Microscope and analyzed using ImageJ and GraphPad Prism software.

### Immunoprecipitation (IP) and immunoprecipitation-mass spectrum (IP-MS)

Cells were lysed on ice for 30 min using IP lysis buffer (Beyotime, Cat#P0037). After centrifugation at 15,000 rpm for 30 min, the supernatant containing 1 mg of protein was incubated with 1µg of antibody overnight at 4 °C with rotation. Protein A/G Magnetic Beads (MCE, Cat#HY-K0202) were then added and incubated for 1 h at 4 °C with rotation. The beads were washed four times with IP buffer, and 2 × SDS loading buffer was added, followed by boiling before immunoblotting detection. For IP-MS, the prepared samples were sent to Shanghai APTBIO Biotechnology Co., Ltd. for mass spectrometry detection.

### CUT&Tag qPCR

CUT&Tag qPCR was performed using the Hyperactive Universal CUT&Tag Assay Kit for Illumina Pro (Vazyme, Cat#TD904), following the manufacturer’s instructions. In brief, HepG2-TP53-KO, SK-hep-1 and LO2 cells transfected with the target plasmid were immobilized on concanavalin A-coated magnetic beads. After resuspension in antibody buffer, cells were sequentially incubated with anti-Flag and IgG primary and secondary antibodies. The pA/G-Tnp Pro transposase was added to the samples for binding after secondary antibody incubation. Following transposon activation and tag mentation, genomic DNA was extracted and Stop Buffer (Vazyme, Cat#TD904-C1) was added. The enrichment and specificity of the targeted DNA fragments were finally evaluated by qPCR and the primers were listed in Table S1.

### Bioinformatics and statistical analysis

All datasets used in this study are publicly accessible, with detailed information provided in Table S2. The normality of the data distribution was confirmed using the Shapiro-Wilk test, and the homogeneity of variances was evaluated via Levene’s test. Student’s t-test (2-tailed) was employed to assess intergroup differences between two cohorts, while one-way ANOVA and two-way ANOVA were applied to evaluate the variations across multiple groups. Data were presented as means ± standard deviation (SD) or means ± standard error of the mean (SEM). Categorical data were analyzed using chi-squared tests. All statistical data were calculated using the GraphPad Prism 7 (GraphPad Software Inc., La Jolla, CA, USA). Statistical significance was designated at *P* < 0.05 (*), *P* < 0.01 (**), and *P* < 0.001 (***), while ns indicated non-significant differences.

## Results

### *DCTN2*, a cellular senescence associated gene, is up-regulated in hepatocellular carcinoma

Cellular senescence plays “double-edged sword” roles in hepatocellular carcinoma progression through inducing persistent tumor cell proliferation arrest and senescence-associated secretory phenotype 19. To further investigate the key genes involved in cellular senescence of liver cancer, integrative bioinformatic profiling was applied to identify the crucial genes in HCC datasets. First, we examined four independent public transcriptomic datasets of hepatocellular carcinoma (GSE76427, GSE36376, GSE325097 and TCGA-LIHC) and delineated 132 differentially expressed genes (DEGs) in HCC (HCC DEGs); then, 20 shared HCC senescence-associated genes (HCC SAGs) were identified through interrogating HCC DEGs above and two cellular senescence associated datasets (extracted from ADEIP database and GSE63577); Moreover, Cox proportional hazards model analysis revealed that the expression of dynactin subunit 2 (*DCTN2*) predicted the worst overall survival of HCC patients among 20 HCC SAGs, therefore, we focused on DCTN2 for further investigation (**Figure S1A**).Under physiological conditions, DCTN2 expression gradually decreased in human liver and blood tissues with advancing age from ADEIP platform 20 (**Figure S1B-C**). Nevertheless, DCTN2 expression unanimously and significantly increased with disease progression across multiple HCC datasets from the TCGA database and the Gene Expression Omnibus (GEO) repository (**Figure 1A-C&S1D**), which indicated that *DCTN2* might play important roles in cellular senescence of HCC. Consistently, Our findings indicated significantly increased mRNA and protein expression of DCTN2 in HCC cell lines (Huh-7, MHCC97H, SK-hep-1, HepG2, SNU-182 and Hep3B) compared to normal liver cell lines (THLE-2 and LO2) through RT-qPCR and Western blot assays (**Figure 1D**). To further validate the expression of DCTN2, we extracted the spatial transcriptomics data and single-cell RNA sequencing results from Integrative Molecular Database of Hepatocellular Carcinoma (HCCDBV2) and GSE166635, and found that DCTN2 expression of tumor cells was significantly elevated compared to other cell types including normal, stromal and immune cells in HCC microenvironment (**Figure 1E&S1E-F**) 21. To elucidate the underlying mechanism leading to DCTN2 upregulation in HCC, we investigated the association between the mutational status of well-known driver genes and *DCTN2* expression using HCC datasets from the cBioPortal database and Comprehensive Analysis on Multi-Omics of Immunotherapy in Pan-cancer (CAMOIP, a tool for analyzing the expression data and mutation data from the TCGA) 22.

We found that *DCTN2* genomic alterations across independent HCC cohorts with the highest frequency (>1.5%) detected in the TCGA Firehose Legacy cohort, and alteration frequency suggested that gene amplification might increase the expression of *DCTN2* in liver cancer (**Figure S1G**). Interestingly, we detected a significant positive association between *DCTN2* expression and TP53 mutational status. Consistently, *DCTN2* expression was markedly elevated in the TP53-mutant group relative to the wild-type group within the TCGA dataset (**Figure 1F&S1H**). Furthermore, chromatin immunoprecipitation (ChIP) results from HepG2 cells in the ENCODE project 23 showed the binding of TP53 to the DCTN2 promoter, which indicated that p53 may transcriptionally regulate DCTN2 in HCC (**Figure S1I**). To verify this hypothesis, we overexpressed wild-type TP53 and a series of common TP53 mutants (R175H, R248W, R249S and R273H) 24 in HepG2 TP53-KO, LO2, and SK-hep-1cells, and demonstrated that wild-type TP53 exerted suppressive regulation on DCTN2. Conversely, ectopic expression of mutant TP53 lost this suppressive regulation, leading to recovered DCTN2 mRNA and protein abundance relative to wild-type TP53, which was consistent with the phenotype that DCTN2 expression in HepG2, SK-hep-1, and SNU-182 (TP53 wild-type cell lines) was lower than that in Huh-7, MHCC97H and Hep3B (TP53 mutant cell lines) (**Figure 1D, 1G-H&S1J-K, S1N**). Moreover, dual-luciferase reporter studies suggested that TP53 could directly bind to the *DCTN2* gene promoter and TP53 mutants elevated DCTN2 expression, which was confirmed by Cut&Tag and qPCR assay (**Figure 1I-K&S1L-M**). Collectively, we revealed that TP53 mutation led to DCTN2 upregulation primarily through loss of WT p53-mediated transcriptional repression and DCTN2 could be a putative cellular senescence regulator in HCC.

### DCTN2 promotes HCC cell proliferation *in vitro* and *in vivo*

To determine whether DCTN2 regulated the HCC malignant phenotype, we first examined its impact on cell proliferation. Considering the elevated expression of DCTN2 in HCC cell lines, we knocked down DCTN2 expression in MHCC97H and SK-hep-1 cell lines, and the depletion efficiency was verified by RT-qPCR and western blot assays (**Figure 2A&S2A**). As expected, growth curve, colony formation and bromodeoxyuridine (BrdU)-incorporation assays indicated that DCTN2-depleted HCC cells showed dramatically reduced proliferative ability (**Figure 2B-E&S2B-E**). Notably, overexpression of DCTN2 markedly promoted cell proliferation in MHCC97H and SK-hep-1 *in vitro* (**Figure 2F-J&S2F-J**). We then detected the cell cycle progression via flow cytometry. DCTN2 knockdown elevated G0/G1 populations in both cell lines, mirroring the cell cycle arrest phenotype of cellular senescence (**Figure S2K-N**). Consistently, Cyclin D1 and CDK4 protein levels were obviously decreased in the DCTN2 knockdown group versus the controls, two core proteins governing the G0/G1 checkpoints (**Figure S2O**). Besides, we analyzed the effect of DCTN2 knockdown on cell apoptosis and found that silencing of DCTN2 could not induce apoptosis in MHCC97H and SK-hep-1 cells, as confirmed by both Annexin V/PI flow cytometry and immunoblotting for cleaved-PARP (**Figure S2P-Q**). To determine whether DCTN2 exerts indispensable effects on HCC proliferation *in vivo*, subcutaneous xenograft tumors were generated within nude mice. The results demonstrated that knockdown of DCTN2 remarkably inhibited xenograft tumor formation, along with reduced tumor volume and weight versus controls (**Figure 2K-N**). Moreover, less Ki67 positive staining was observed in the xenograft tumors from DCTN2 knockdown groups compared to control group through immunohistochemical (IHC) assay (**Figure 2O-P**), which demonstrated that DCTN2 enhanced HCC proliferation *in vivo*.

### DCTN2 enhances HCC cell migration and angiogenesis

To explore the potential role of DCTN2 in HCC metastatic process, we performed wound-healing and trans-well assays and revealed that knockdown of DCTN2 dramatically inhibited HCC cell migration and invasion (**Figure 3A-C&S3A-C**). On the contrary, we showed that DCTN2 overexpression significantly promoted cell migration and invasion of MHCC97H and SK-hep-1 through up-regulating SLUG, SNAIL, N-cadherin, ZEB1, ZEB2 and TWIST1 expression levels, while downregulating E-cadherin expression level *in vitro* (**Figure 3D-F, 3K&S3D-F, S3J**). Moreover, cellular morphological observation demonstrated that DCTN2 depletion obviously suppressed the epithelial-mesenchymal transition (EMT) process, which was validated by phalloidin immunofluorescence staining and gene ontology (GO) enrichment analysis from RNA-seq data of DCTN2 knockdown in HCC cells (**Figure 3G-H&S3G, S5E**). Numerous studies have reported that tumor cell senescence suppressed tumor metastasis by inhibiting angiogenesis 25, 26. To determine whether DCTN2 modulates this process, we first conducted RT-qPCR in HCC cells, which revealed that DCTN2 knockdown markedly reduced mRNA levels of ANGPT2, CXCL8, MMP2, MMP9 and MMP1, whereas DCTN2 overexpression significantly upregulated these pro-angiogenic mediators (**Figure 3I-J&S3H-I**). We then collected CM from DCTN2-overexpressing or knockdown HCC cells and corresponding control groups for incubation with HUVECs, from which we provided evidence showing that DCTN2 knockdown in HCC cells could remarkably repress, while ‌ectopic expression of DCTN2 enhanced, HUVECs tube formation ability (**Figure 3L-M&S3K-L**). Furthermore, using a CXCL8 neutralizing antibody partially rescued the pro-tube formation phenotype and enhanced migratory capacity driven by DCTN2 overexpression (**Figure 3M, 3O-P&S3L**). Consistently, reduction of CD31 IHC staining in xenograft tumors compared to control groups further confirmed that DCTN2 knockdown significantly inhibited tumor angiogenesis *in vivo* (**Figure 3N**). These results suggested that DCTN2 boosted HCC cell migration and angiogenesis.

### DCTN2 inhibits cellular senescence in HCC progression

Based on these findings above, we assumed that DCTN2 acted as a cellular senescence suppressor to promote HCC malignant progression. To verify this assumption, we developed two cellular senescence models in HCC cells treated with H_2_O_2_ and cisplatin as previously reported 27. In line with the down-regulation of DCTN2 expression in older adults, H_2_O_2_ and cisplatin treatment significantly decreased DCTN2 expression in MHCC97H and SK-hep-1 (**Figure 4A&S4A**). SA-β-Gal staining assay demonstrated that inhibition of DCTN2 led to cellular senescence, while overexpression of DCTN2 could restore H_2_O_2_ and cisplatin induced the senescence phenotype in HCC cells (**Figure 4B-E&S4B-E**). Meanwhile, knockdown of DCTN2 in MHCC97H and SK-hep-1 cells upregulated the expression of senescence markers including γ-H2AX, p21, p27 and p16, reduced Lamin B1 levels, and suppressed proliferation-related p-Rb and Ki67 expression. Conversely, overexpression of DCTN2 resulted in entirely opposite expression trends (**Figure 4F&S4F**)‌. Furthermore, RT-qPCR results indicated that multiple senescence-associated secretory phenotype genes were notably elevated in DCTN2 knockdown HCC cells compared to control groups, such as IL-6, IL-1A and TNFα (**Figure 4G&S4G**). Importantly, the enhanced cellular senescence evidenced by IHC staining of senescence-related marker proteins was detected in DCTN2 knockdown xenograft tumor tissues compared to the control groups *in vivo* (**Figure 4H-I**), supporting the hypothesis that DCTN2 inhibited cellular senescence in HCC progression.

### DCTN2 attenuates Hippo signaling pathway

To decipher the underlying mechanism of DCTN2 for inhibiting cellular senescence while promoting HCC progression, whole-transcriptome RNA-seq analysis was performed to characterize downstream signaling networks, identifying 435 differentially expressed genes (|Log_2_FC| > 0.58, *P <* 0.05) in DCTN2-depleted cells compared to control groups (**Figure 5A**). To verify the transcriptomic data, we selected two significantly altered genes-*BMP7* (HCC metastasis-associated factor) and *CCND2* (cell cycle G1/S transition regulator) 28, 29 for experimental validation and proved that DCTN2 genetic perturbation modulated both genes mRNA levels by RT-qPCR assay, concordant with the RNA-seq results (**Figure S5A-D**). Meanwhile, we found that DCTN2 was involved in cellular senescence process through Kyoto Encyclopedia of Genes and Genomes (KEGG) pathway analysis and Gene Set Enrichment Analysis (GSEA) (**Figure 5B&S5E-F**). Interestingly, KEGG analysis suggested that DCTN2 may regulate Hippo signaling pathway (**Figure 5B**). To date, accumulating studies demonstrated the essential function of the Hippo signaling pathway in modulating tumor cellular senescence, such as LATS1/2, MST1/2, and YAP/TAZ, which regulated the expression of multiple SASP components including various interleukins and chemokines 16, 30-32, therefore we hypothesized that DCTN2 suppressed senescence by regulating the Hippo signaling pathway in HCC cells. In our study, immunoblotting assays showed that DCTN2 depletion enhanced phosphorylation levels of MST1/2, LATS1/2, and YAP/TAZ normalized to respective total proteins, while ectopic expression reciprocally attenuated these effects in MHCC97H and SK-hep-1 (**Figure 5C&S5G**). Immunofluorescence results indicated that DCTN2 silencing reduced the nuclear localization of YAP, while the overexpression of DCTN2 increased the nuclear enrichment of YAP in HCC cells, which was validated by the western blot results from nucleus-cytoplasm fractionation assay upon DCTN2 knockdown and overexpressing HCC cells (**Figure 5D-E&S5H-I**). As CTGF and CYR61 were two key target genes of the Hippo signaling pathway, we examined these two protein levels in our study and found that knockdown of DCTN2 significantly inhibited CTGF and CYR61 expression, whereas its overexpression reciprocally upregulated their expression; this phenotype was confirmed by the positive correlation analysis between DCTN2 expression and CTGF or CYR61 expression extracted from the TCGA-LIHC dataset (**Figure 5C, 5F-G&S5G**). More importantly, ‌YAP activator PY60 and constitutively active YAP (YAP^5SA^) overexpression obviously restored DCTN2-silencing phenotype in HCC cells, abrogating the anti-proliferative/migratory effects while counteracting senescence induction in MHCC97H and SK-hep-1 (**Figure 5H-O&S5J-Q**). Taken together, DCTN2 exacerbated HCC progression through inhibiting the Hippo signaling pathway.

### DCTN2 inhibits the Hippo signaling pathway through interacting with TRIM21 in HCC

To delineate the molecular mechanism underlying DCTN2-mediated repression of the Hippo signaling pathway in HCC, we conducted IP-MS to screen putative proteins interacting with DCTN2. In DCTN2-overexpressing SK-hep-1 cells, we identified tripartite motif-containing 21 (TRIM21), a RING-type E3 ubiquitin ligase critical for protein homeostasis, as a novel binding protein of DCTN2 (**Figure 6A-B**). Subsequently, we performed exogenous and endogenous co-immunoprecipitation (Co-IP) assays to confirm the interaction between DCTN2 and TRIM21 in HEK-293T, MHCC97H and SK-hep-1, which were evidenced by the immunofluorescence staining results that DCTN2 predominantly co-localized with TRIM21 in HCC cells (**Figure 6C-D&S6A-B**). It has been reported that TRIM21 regulated Hippo signaling pathway 33, 34. Thus, we proposed that DCTN2 might interact with TRIM21 to regulate Hippo signaling pathway in HCC.

Growing evidence indicated that TRIM21 both promoted and inhibited carcinogenesis across diverse cancer contexts, such as TRIM21 overexpression, which inhibited renal carcinoma malignancy while enhanced pancreatic cancer growth ability 35-37. Therefore, we further examined the roles of TRIM21 within HCC context. We found that TRIM21 expression was markedly decreased in HCC lines and clinical samples compared to normal liver cell lines and tissues (**Figure 7A&S6C**). In addition, growth curve, colony formation, BrdU incorporation, wound healing, trans-well and SA-β-Gal staining assays indicated that ectopic expression of TRIM21 attenuated cell growth and migration, and triggered cellular senescence in HCC cells, suggesting TRIM21 acted as a tumor suppressor of HCC progression in our study (**Figure S6D-P**).

TRIM21 was reported to ubiquitinate MST2 with K63-linked polyubiquitin chains and to increase MST2 homodimerization and kinase activity or ubiquitination mediated degradation of MST1 in tumors 33, 34. However, our findings revealed that TRIM21 significantly upregulated, whereas DCTN2 markedly downregulated, the phosphorylation of MST2 in HCC cells, without affecting the basal protein levels of MST1 or MST2 (**Figure 5C&S5G, S6Q**). To clarify the regulatory mechanism of vilazodone in HCC, we performed Co-IP and ubiquitination assays, which confirmed that vilazodone strengthened the endogenous TRIM21-MST2 binding and drove TRIM21-dependent K63-linked ubiquitination of MST2 (**Figure S6R-T**). Additionally, ectopic TRIM21 overexpression rescued the decreased p-MST1/2 levels induced by DCTN2 overexpression in both MHCC97H and SK-hep-1 cells (**Figure S6U**); therefore, we focused on the regulatory roles of DCTN2 and TRIM21 in MST2 phosphorylation. As anticipated, overexpression of DCTN2 decreased while DCTN2 silencing elevated the MST2 ubiquitination in HEK-293T (**Figure 6E-F**). Interestingly, we also observed that DCTN2 overexpression down-regulated, while DCTN2 inhibition increased the K63-linked ubiquitination of MST2, rather than K48-linked ubiquitination (**Figure 6G-H&S6V-W**). In addition, DCTN2 overexpression ‌markedly disrupted MST2 homodimer formation, ‌whereas DCTN2 depletion ‌promoted dimer assembly in HEK-293T (**Figure 6I-J**), in line with prior study of TRIM21 in colorectal cancer 34. Notably, in both MHCC97H and SK-hep-1 cell lines, neither overexpression nor knockdown of DCTN2 modulated TRIM21 expression, indicating DCTN2 inhibited Hippo signaling pathway was independent on TRIM21 protein level (**Figure 6K&S6X**). We performed Co-IP assays and proved that the interaction between exogenously tagged MST2-Myc and TRIM21-HA was obviously reduced by ectopic expression of DCTN2-Myc in HEK-293T, while knockdown groups showed the opposite effect compared to control groups (**Figure 6L-M**). More importantly, the same results regarding the competition between DCTN2 and TRIM21 for binding to MST2 were also validated in MHCC97H and SK-hep-1 (**Figure 6N&S6Y**). Collectively, these results indicated that DCTN2 competed with TRIM21 to decrease K63-linked ubiquitination of MST2, thereby inhibiting MST2 homodimer formation and subsequently attenuating Hippo signaling pathway in HCC.

### DCTN2 expression is negatively correlated with clinical outcome in HCC

Consistent with the expression levels in HCC cells, we validated that the protein expression of DCTN2 was dramatically up-regulated in tumors compared to paired normal tissues, while the expression of TRIM21 and p-MST1/2 was decreased (**Figure 7A-B**). Notably, DCTN2 expression was negatively correlated with p-MST1/2 expression in HCC tumor tissues (**Figure 7B**). We examined the TCGA dataset of HCC and revealed remarkable increase of *DCTN2* mRNA levels in HCC patients with high pathological grades and high Alpha-Fetoprotein (AFP) concentration (**Figure 7C-D**). Furthermore, analysis of TCGA and KM plotter datasets 38 revealed that patients with higher DCTN2 expression had worse overall survival (OS). Poor disease-specific survival (DSS) and progression-free interval (PFI) were also observed. Receiver operating characteristic curve (ROC) analysis demonstrated that DCTN2 served as a promising prognostic biomarker for HCC (AUC = 0.977), compared to other validated markers including AFP (AUC = 0.723), ZEB1 (AUC = 0.682) and GPC3 (AUC = 0.919) 39-41 (**Figure 7E-G**). In line with public datasets findings, we performed IHC analysis in cancerous TMA and revealed DCTN2 expression was obviously elevated in cancer tissues compared to paired normal tissues (**Figure 7H-I**). More importantly, patients with lower DCTN2 expression had prolonged overall survival, better progression free survival (PFS) and lower recurrence rate compared to those with elevated DCTN2 expression in TMA dataset (**Figure 7J**). These results indicated that the DCTN2-TRIM21-Hippo signaling axis played crucial roles in HCC progression.

### Targeting TRIM21 with vilazodone potentiates sorafenib anticancer efficacy in HCC

To test the potential clinical value of DCTN2-TRIM21-Hippo signaling axis in HCC treatment, we screened drugs targeting DCTN2-TRIM21-Hippo signaling axis to inhibit HCC progression. Because no DCTN2-targeting inhibitors have been reported, we focused on TRIM21 targets. Recent study had indicated that vilazodone, an antidepressant indicated for the treatment of major depressive disorder, directly bound to TRIM21 and enhanced the interaction of TRIM21 with MST2 to suppress invasion and metastasis of colorectal cancer 34, whereas its therapeutic efficacy was not examined in HCC. Due to the influence of tumor heterogeneity, the regulatory effect of vilazodone on TRIM21-MST2 binding was further validated in HCC, as shown in **Figure S6R-T**. Furthermore, even as the first proved targeted drug, sorafenib resistance presents a formidable challenge, and sorafenib initiates HCC cellular senescence by regulating cleavage of p62 42. Therefore, we further assessed the therapeutic potential of vilazodone and explored whether vilazodone could synergize with sorafenib in HCC. We treated MHCC97H and SK-hep-1 with vilazodone and sorafenib‌, and proved that vilazodone and sorafenib both significantly inhibited hepatocellular carcinoma cell proliferation and migration, with their combination demonstrating enhanced antitumor efficacy *in vitro* (**Figure 8A-H&S7A-H**). Consistently, SA-β-Gal staining confirmed that vilazodone enhanced sorafenib-induced senescence in HCC cells, and this effect was further corroborated by immunoblotting assay (**Figure 8I-K&S7I-K**). In addition, we revealed that vilazodone treatment significantly impeded tumor growth and promoted senescence process *in vivo* compared to vehicle control treatment, and synergized with sorafenib treatment for HCC, which were evidenced by Chou-Talalay CI analysis (all CI values < 0.9), obvious reduction of xenograft tumor volumes and masses, and the examination of their weights, key organs and tissues by H&E staining did not reveal significant toxic side effects (**Figure 8L-N&S7L-M**). IHC staining indicated that vilazodone treatment increased TRIM21, p16, p21, γ-H2AX and p-MST2 positive cells, while it alone or synergized with sorafenib decreased Ki67 positive tumor cells compared to reciprocal control group in xenograft tumor tissues (**Figure 8O-V**). Collectively, these results suggested that vilazodone exerted an agonist of TRIM21, highlighting its potential as a novel chemotherapeutic agent for HCC treatment.

## Discussion

Cellular senescence is a process in which cells cease proliferation and maintain a stable state in response to various stresses, supporting organismal homeostasis. Currently, cancer therapies are expected to combine approaches that promote senescence with conventional treatments, potentially enhancing the efficacy 43, 44. In this study, to decipher the underlying mechanism of HCC cellular senescence, we screened and identified DCTN2 as an oncogene in HCC via repressing Hippo signaling pathway. We found that DCTN2 was dramatically up-regulated in HCC progression, which was regulated by TP53 gene mutation. We demonstrated that DCTN2 significantly inhibited cellular senescence, thereby promoting cell proliferation, migration, invasion, and angiogenesis. Conversely, knockdown of DCTN2 exhibited an opposing tumor-suppressive role in HCC. Mechanistically, DCTN2 repressed cellular senescence through its interaction with TRIM21, which decreased K63-linked polyubiquitination of MST2 and ultimately attenuating the Hippo signaling pathway. Elevated DCTN2 expression was positively correlated with poor prognosis in HCC patients. Additionally, combined targeting of TRIM21 using vilazodone and sorafenib synergistically induced cellular senescence in HCC cells, as demonstrated in both *in vitro* and *in vivo* models.

DCTN2 is a member of the dynactin family and participates in several cellular activities, such as mitosis, intracellular trafficking, and stress responses, and is also vital for synapse stability and the proper functioning of neurons 45, 46. Recent studies indicated that dynactin, encoded by *DCTN2*, played a role in facilitating axonal growth and the transport of multiple organelles through a pathway involving phosphatidylinositol 3-kinase catalytic subunit type 3, ankyrin-B, and dynactin 47. While the role of dynactin in cancer had received widespread attention, the function of DCTN2 in tumors was limited, with its specific contribution to liver cancer cell senescence still insufficiently explored. In this study, we found that DCTN2 might be upregulated by TP53 mutation and gene amplification in HCC and negatively correlated with the survival rate of HCC patients. Inhibition of DCTN2 promoted HCC cellular senescence, thereby inhibiting tumor proliferation and migration alongside cell cycle arrest. Additionally, RNA-seq results indicated that DCTN2 enhanced HCC malignancy by inhibiting the Hippo signaling pathway. Moreover, IP-MS results indicated that DCTN2 interacted with TRIM21, which was validated by Co-IP and immunofluorescence assays.

As an important E3 ubiquitin ligase member of the TRIM family, TRIM21 is involved in the regulation of biological processes, such as autophagy, immunity, and carcinogenesis 48. Nevertheless, accumulating studies indicated that the function of TRIM21 in tumors varied across different tumor types. For example, in renal cell carcinoma, TRIM21 degraded the sterol regulatory element binding transcription factor 1 (SREBF1) via ubiquitination and decreased the lipid contents, inhibiting renal cell carcinoma lipid accumulation and growth 36. A recent study reported that TRIM21 promoted tumor growth and gemcitabine resistance in pancreatic cancer by inhibiting arachidonic acid metabolism 35. Moreover, various studies suggested that TRIM21 might play different roles even in hepatocellular carcinoma. Recent study indicated that TRIM21 interacted with and ubiquitinated MST1, resulting in MST1 degradation and YAP activation, thereby enhancing cell growth and metastasis in HCC 33. However, another article demonstrated that TRIM21 facilitated the proteasomal degradation of reticulophagy regulator 1 (RETREG1), suppressing the AKT signaling pathway and HCC progression 49. In a high-fat and high-cholesterol diet induced murine HCC model, TRIM21 deficiency increased HCC carcinogenesis in a non-alcoholic steatohepatitis context 50. Similarly, TRIM21 functioned as a tumor suppressor by binding to and ubiquitinating other key proteins, thereby regulating their stability in HCC, such as fatty acid synthase (FASN), nucleolin (NCL), receptor interacting serine/threonine kinase 1 (RIPK1), apolipoprotein E (ApoE) and vacuolar protein sorting 72 homologue (VPS72) 51-55. Therefore, to further elucidate the function of TRIM21 in HCC cellular senescence and progression, we examined its expression in HCC cell lines and tissues and found that TRIM21 was significantly downregulated in HCC cells and tissues compared to corresponding control groups. More importantly, overexpression of TRIM21 obviously inhibited cell proliferation, migration and promoted cellular senescence in MHCC97H and SK-hep-1, which indicated that TRIM21 acted as inhibitory roles of HCC malignance in our study, which was consistent with previous studies 56, 57.

Considering the observation that TRIM21 overexpression didn’t decrease the basal total protein levels of MST1 and MST2 in MHCC97H and SK-hep-1, we hypothesized that DCTN2 might interact with TRIM21 and restrain the Hippo signaling pathway through regulating TRIM21-mediated K63-linked ubiquitination of MST2. As expected, ectopic expression of DCTN2 sufficiently inhibited MST2 ubiquitination, oligomerization and autophosphorylation, which was significantly reversed by TRIM21 overexpression. Besides, DCTN2 didn’t regulate TRIM21 protein expression. Mechanistically, we demonstrated that DCTN2 repressed cellular senescence by competing with TRIM21 to increase MST2 K63-linked ubiquitination and inhibit its dimerization and autophosphorylation, rather than regulating MST1 or MST2 expression. Nevertheless, although no inhibitors targeting DCTN2 were documented to date, recent research revealed that vilazodone directly bound to TRIM21 and potentiated its E3 ligase activity, thereby enhancing ubiquitination of MST2 to activate the Hippo signaling pathway and exert effective anti-metastatic effects in colorectal cancer 34; therefore, we selected vilazodone for the follow-up study. In line with the previous findings in colorectal cancer, we found that vilazodone markedly suppressed cell growth, migration and invasion, and triggered cellular senescence in HCC. Moreover, vilazodone targeting TRIM21 significantly improved the efficacy of sorafenib *in vitro* and *in vivo*, and did not cause significant adverse effects on body weight or the morphology of major organs in mice.

In conclusion, our results indicated that DCTN2 competed with TRIM21 to ubiquitinate MST2 and restrained the Hippo signaling pathway, thereby repressing cellular senescence in HCC progression. Moreover, we proved that inhibition of DCTN2/TRIM21/MST2 signaling axis via vilazodone repurposing combined with sorafenib treatment might provide novel clinical treatment strategies for HCC in the future.

## Supplementary Material

Supplementary figures and tables.

Supplementary data.

## Figures and Tables

**Figure 1:**
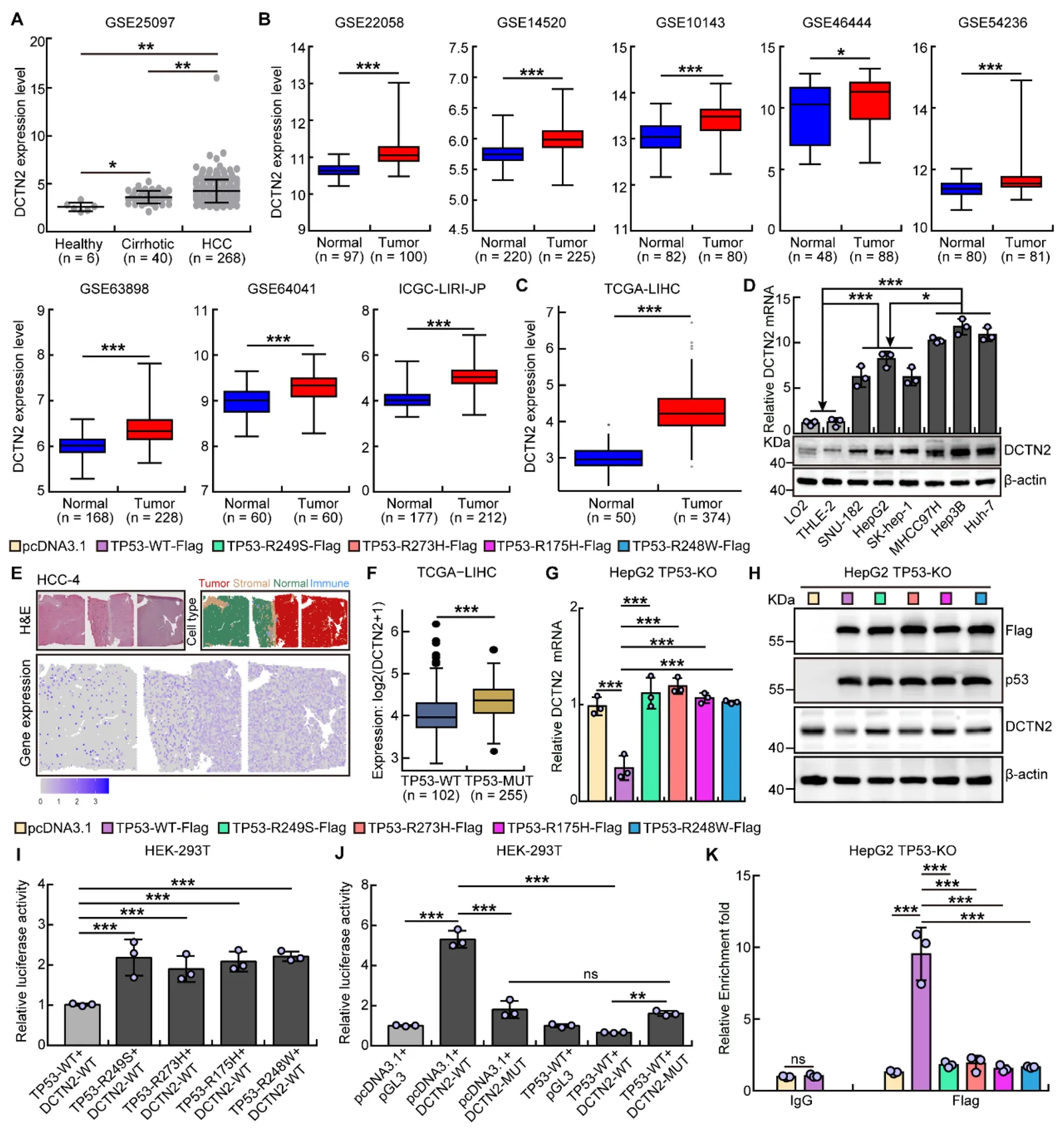
** TP53 mutation increased the expression of DCTN2 in HCC. (A)** *DCTN2* expression increased progressively from healthy controls (n = 6) to cirrhotic patients (n = 40) and HCC patients (n = 268).** (B)** Eight independent datasets extracted from online GEO database confirmed that DCTN2 was highly expressed in HCC tissues versus normal liver tissues. **(C)** *DCTN2* expression levels in HCC versus matched normal liver tissues from the TCGA cohort. **(D)** The relative expression level of DCTN2 in HCC cell lines examined by RT-qPCR (top) and immunoblotting (bottom). The normal human hepatic epithelial cell lines LO2 and THLE-2 served as control. **(E)** Spatial transcriptomic analysis of a HCC tissue section from HCCDB V2 revealed the spatial expression distribution of *DCTN2*, paired with H&E staining and cell-type annotation (Tumor, Stromal, Normal, Immune). **(F)** DCTN2 expression was significantly higher in TP53-mutant HCC tumors than in wild-type tumors from TCGA-LIHC dataset. **(G-H)** DCTN2 expression levels were examined by RT-qPCR (G) and immunoblot (H) in HepG2 TP53-KO cells transiently transfected with pcDNA3.1, TP53-WT-Flag, or TP53 common mutants (R249S, R273H, R175H and R248W).** (I)** The TP53 mutants (R249S, R273H, R175H and R248W) significantly enhanced the transcriptional activity of DCTN2 promoter compared with wild-type TP53, as detected by dual luciferase reporter assays (n = 3). **(J)** The dual luciferase reporter assay was performed to detect the effect of the DCTN2 promoter mutant on the activation of the *DCTN2* gene promoter by TP53 wild-type (n = 3). **(K)** CUT&Tag qPCR assay verified significant enrichment of Flag-tagged TP53 at the DCTN2 locus in HepG2 TP53-KO cells, whereas mutants TP53 impeded this enrichment, IgG served as a negative control. Bars indicate mean ± SD. ns, no significant difference. * P < 0.05, ** P < 0.01, *** P < 0.001.

**Figure 2:**
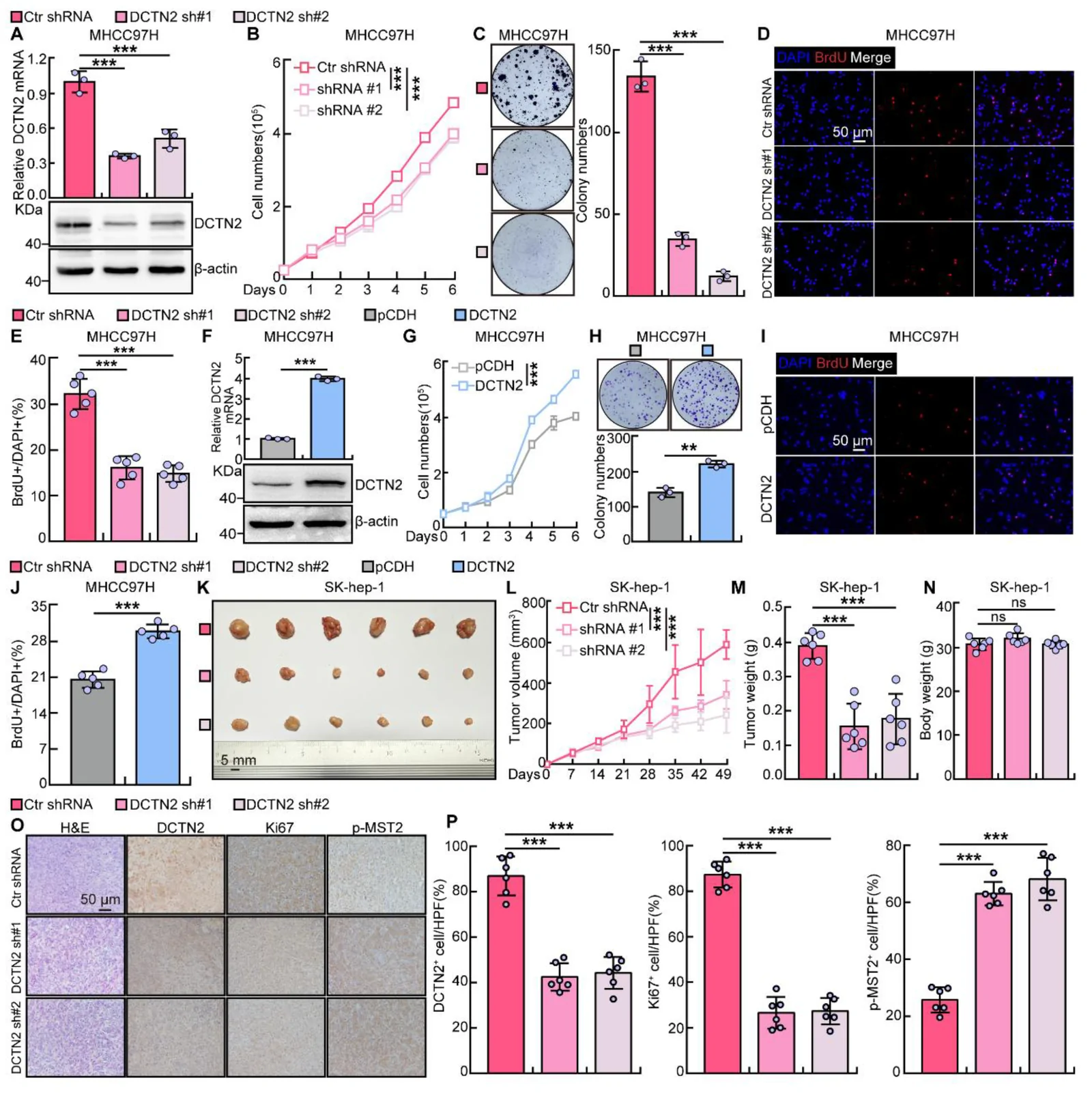
** Depletion of DCTN2 inhibited HCC cell proliferation *in vitro* and *in vivo*. (A)** Establishment of DCTN2-depleted MHCC97H cell lines; knockdown efficiency was verified by RT-qPCR (top) and immunoblotting (bottom). (**B-E**) DCTN2 knockdown led to an obvious reduction of MHCC97H cell proliferation, validated by growth curve assay (B), colony formation experiments (C) and BrdU incorporation assay (D). (E) Quantification data corresponding to panel (D). Scale bar = 50 µm. **(F)** Establishment of DCTN2-overexpressing MHCC97H cell lines, verified by real-time RT-qPCR (top) and immunoblot (bottom).** (G)** Cell growth of the indicated groups was assessed by daily cell counting. **(H)** DCTN2 overexpression notably boosted the colony formation ability of MHCC97H cells, with the statistical quantification of colony numbers presented. **(I-J)** BrdU incorporation assay showed increased BrdU-positive cells in DCTN2-overexpressing MHCC97H cells. (J) Quantitative data corresponding to (I). Scale bar = 50 µm. **(K-N)** DCTN2 ablation markedly repressed xenograft tumor growth: representative tumor images (K), tumor volume measurements (L), data were presented as mean ± SEM, tumor mass quantification (M) and mouse body weight data (N) were presented. **(O-P)** Representative IHC staining images of DCTN2, Ki67 and p-MST2 for indicated xenograft tumors (O), and the quantification results were also indicated (P). Scale bar = 50 µm. Bars indicate mean ± SD. ns, no significant difference. * P < 0.05, ** P < 0.01, *** P < 0.001.

**Figure 3:**
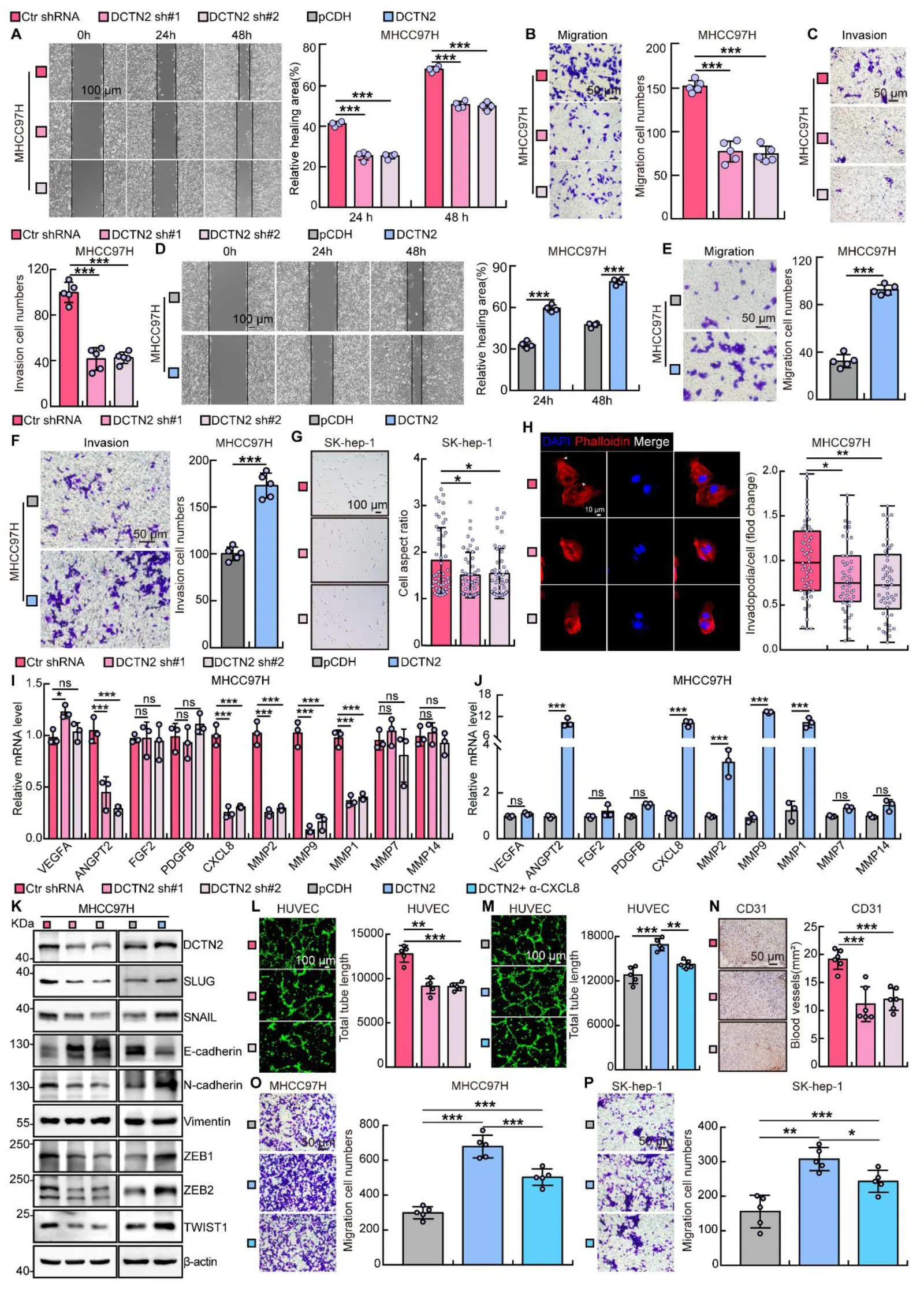
** DCTN2 knockdown inhibited HCC cell migration and angiogenesis. (A-C)** Wound healing (A), trans-well (B), and invasion (C) assays were performed to examine MHCC97H cell migration ability upon DCTN2 knockdown. Scale bars = 100 µm (A), 50 µm (B, C). **(D-F)** MHCC97H cell migration ability upon DCTN2 ectopic expression was examined via wound healing (D), trans-well (E), and invasion (F) assays. **(G)** Representative bright-field images illustrating morphological alterations in SK-Hep-1 cells following DCTN2 knockdown. Scale bar = 100 µm. **(H)** Immunofluorescence staining of phalloidin was performed in indicated cells. White head arrows pointed to the pseudopodia like structure. Scale bar = 10 µm. The corresponding bar chart on the right presented quantitative analysis. (**I-J**) RT-qPCR quantification of pro-angiogenic mediators was conducted in MHCC97H cells with DCTN2 knockdown (I) or overexpression (J).** (K)** The protein expressions of SLUG, SNAIL, E-cadherin, N-cadherin, Vimentin, ZEB1, ZEB2 and TWIST1 were detected in MHCC97H knockdown or overexpression cell extracts. **(L)** The tube formation of primary HUVECs was inhibited by CM collected from MHCC97H cells with DCTN2 inhibition. (**M**) Ectopic DCTN2 overexpression promoted HUVEC angiogenesis, whereas neutralizing CXCL8 (3 μg/mL) partially abrogated this phenotype. Scale bar = 100 µm. **(N)** IHC analysis of CD31 was examined in xenograft tumor tissues. Scale bar = 50 µm. (**O-P**) Ectopic DCTN2 overexpression markedly increased cell migration, while CXCL8 neutralizing antibody partially attenuated this migration ability in MHCC97H (O) and SK-hep-1 (P) cells, the corresponding bar chart on the right presented quantitative analysis. Scale bar = 50 µm. Bars indicate mean ± SD. ns, no significant difference. * P < 0.05, ** P < 0.01, *** P < 0.001.

**Figure 4:**
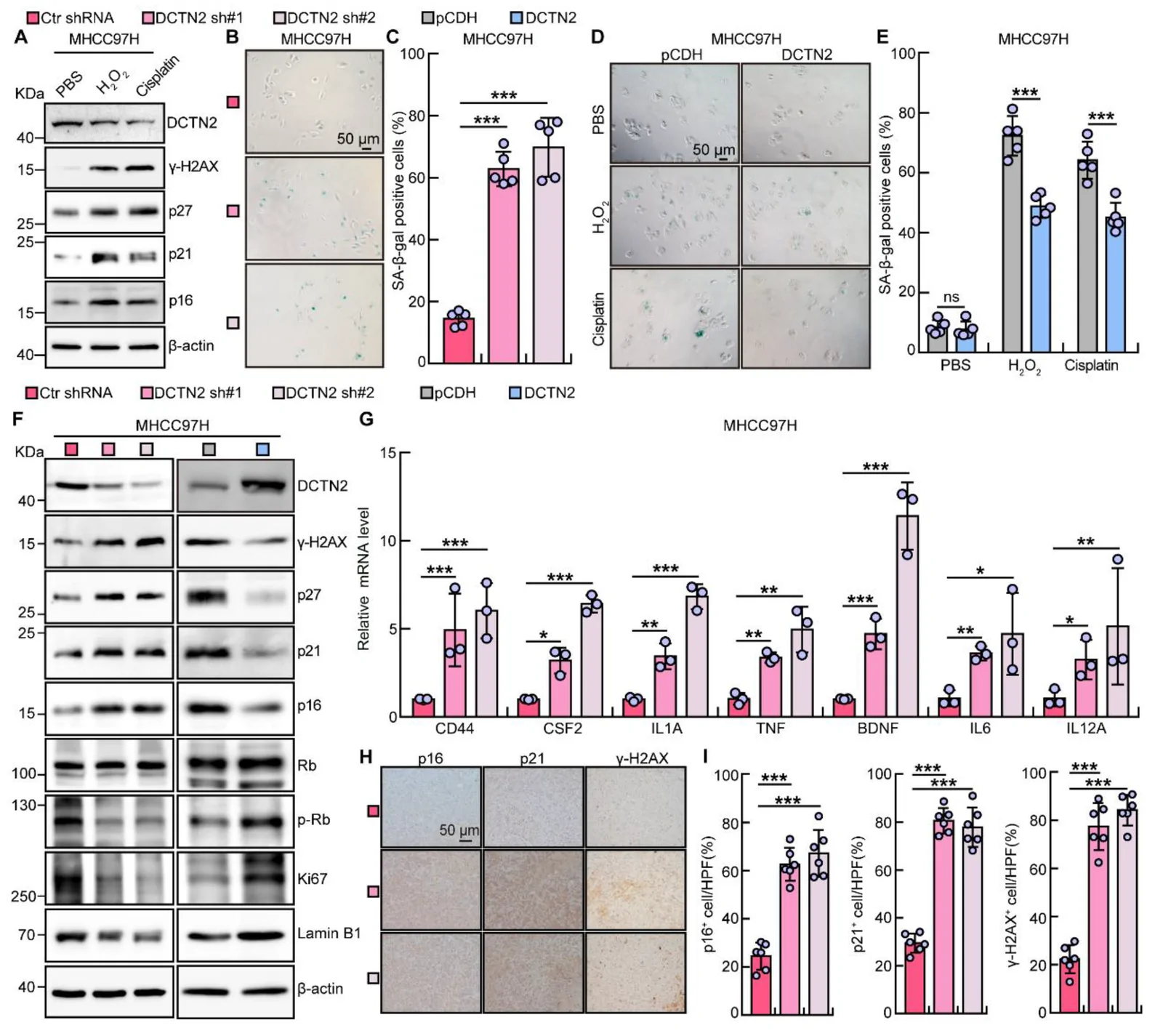
** DCTN2 repressed cellular senescence in HCC. (A)** The protein levels of DCTN2, γ-H2AX, p27, p21 and p16 in MHCC97H cells treated with H₂O₂ or cisplatin were examined using western blot assay. **(B-E)** Changes of SA-β-gal activity were detected in MHCC97H cells with DCTN2 knockdown or overexpression. (C) and (E) were the statistical graphs of (B) and (D), respectively. Data represented the percentage of cells staining positive for SA-β-gal staining. Scale bar = 50µm. **(F)** Immunoblot analysis was performed to assess the expression levels of DCTN2, γ-H2AX, p27, p21, p16, Rb, p-Rb, Ki67 and Lamin B1 in MHCC97H cells with DCTN2 depletion or overexpression. **(G)** The mRNA expression levels of SASP factors including CD44, CSF2, IL-1A, IL-6, IL-12A, TNF and BDNF in DCTN2-depleted MHCC97H cells were detected by RT-qPCR. **(H-I)** Representative IHC staining of p16, p21 and γ-H2AX in nude mouse xenograft tumors of DCTN2-depleted SK-hep-1 cells (H); corresponding quantifications were shown in (I). Scale bar = 50 µm. Bars were the mean value ± SD. ns = no significant. * P < 0.05, ** P < 0.01, *** P < 0.001.

**Figure 5:**
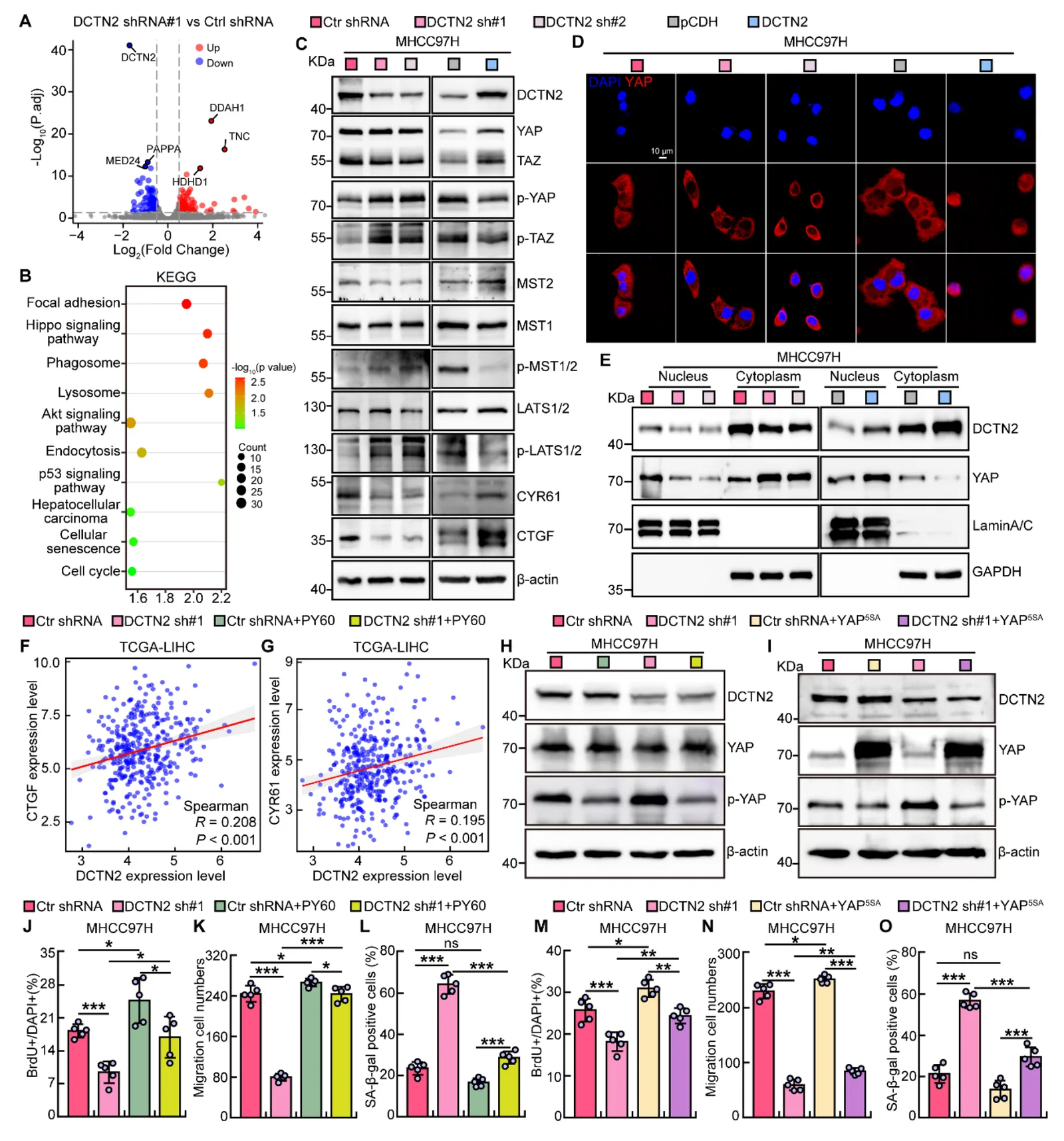
** DCTN2 repressed the Hippo signaling pathway. (A)** RNA-seq analysis was performed on SK-hep-1 cells transfected with DCTN2 shRNA#1, and volcano plots were generated to visualize DEGs. Red, upregulated genes; blue, downregulated genes. (*P <* 0.05; |log₂FC| > 0.58). **(B)** KEGG enrichment analysis of DEGs revealed pathways perturbed upon DCTN2 suppression. **(C)** Western blot analyzed the protein levels of key components in the Hippo signaling pathway from DCTN2 overexpressed or depleted MHCC97H cells, as well as its downstream target genes CYR61 and CTGF. **(D-E)** Immunofluorescence staining (D) and western blot of nucleus-cytoplasm fractionation (E) showed subcellular localization of YAP in the indicated MHCC97H cells. Scale bar = 10 µm. **(F-G)** Correlation analysis between DCTN2 and the Hippo signaling pathway target genes CTGF (F) and CYR61 (G), determined by the TCGA-LIHC dataset. **(H-I)** Immunoblotting assay examined DCTN2, YAP and p-YAP protein levels in the indicated MHCC97H cells. **(J-O)** Treatment with PY60 or ectopic expression of YAP^5SA^ could reverse the phenotypes induced by DCTN2 knockdown, analyzed by BrdU incorporation (J, M), trans-well (K, N) and SA-β-gal staining assays (L, O). Bars indicate mean ± SD. ns, no significant difference. * P < 0.05, ** P < 0.01, *** P < 0.001.

**Figure 6:**
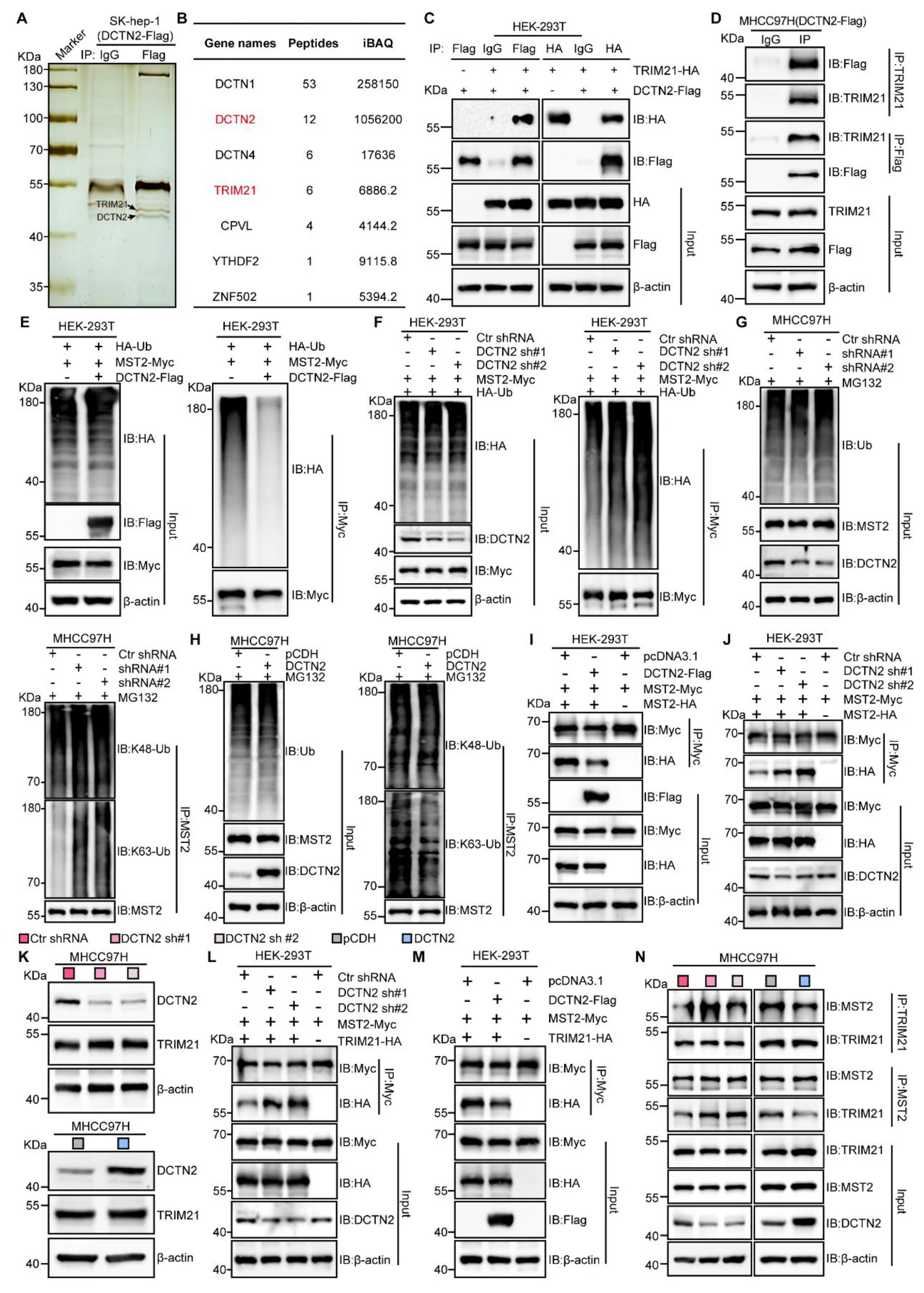
** DCTN2 interacted with TRIM21 and enhanced TRIM21-mediated activation of MST2 in HCC. (A)** Co-IP with Flag antibody in SK-hep-1 cells, subsequent SDS-PAGE silver staining and mass spectrometry; arrows denoted candidate differentially identified proteins. **(B)** Peptide-based ranking of the results shown in (A). **(C-D)** Exogenous (C) and endogenous (D) Co-IP assays were used to clarify the protein-protein interaction between DCTN2 and TRIM21. IP: immunoprecipitation, IB: immunoblotting.** (E-F)** Ubiquitination assays of MST2 in the lysates from DCTN2 overexpressing cells (E) or DCTN2 knockdown cells (F). HEK-293T cells were transfected with the indicated constructs, and cell lysates were subjected to input analysis and IP. **(G)** MHCC97H cells were transfected with DCTN2 depletion construct and treated with MG132. Cell lysates were used for input analysis and IP. IB was performed on MST2 Co-IP samples to detect K48- and K63-linked ubiquitin chains. **(H)** Upregulation of DCTN2 and treated with MG132 in MHCC97H cells, IP was performed with anti-MST2 antibody. Subsequent ubiquitination assay *in vivo* was conducted with antibodies against K48- and K63-linked polyubiquitin chains to analyze the polyubiquitination state of MST2. **(I-J)** Immunoprecipitation of MST2 homodimer in the HEK-293T cells with the ectopic expression of DCTN2 (I) or in the DCTN2 shRNA-transfected HEK-293T cells (J). **(K)** Western blot assay was employed to detect TRIM21 expression in MHCC97H cells subjected to DCTN2 knockdown or overexpression. **(L-M)** Detection of MST2-TRIM21 interaction capacity with DCTN2 shRNA transfection (L) or DCTN2 overexpression (M) in HEK-293T cells.** (N)** Endogenous Co-IP assay of DCTN2 and TRIM21 was used to examine the interactions between DCTN2 and TRIM21 in DCTN2-overexpressed or depleted MHCC97H cells.

**Figure 7:**
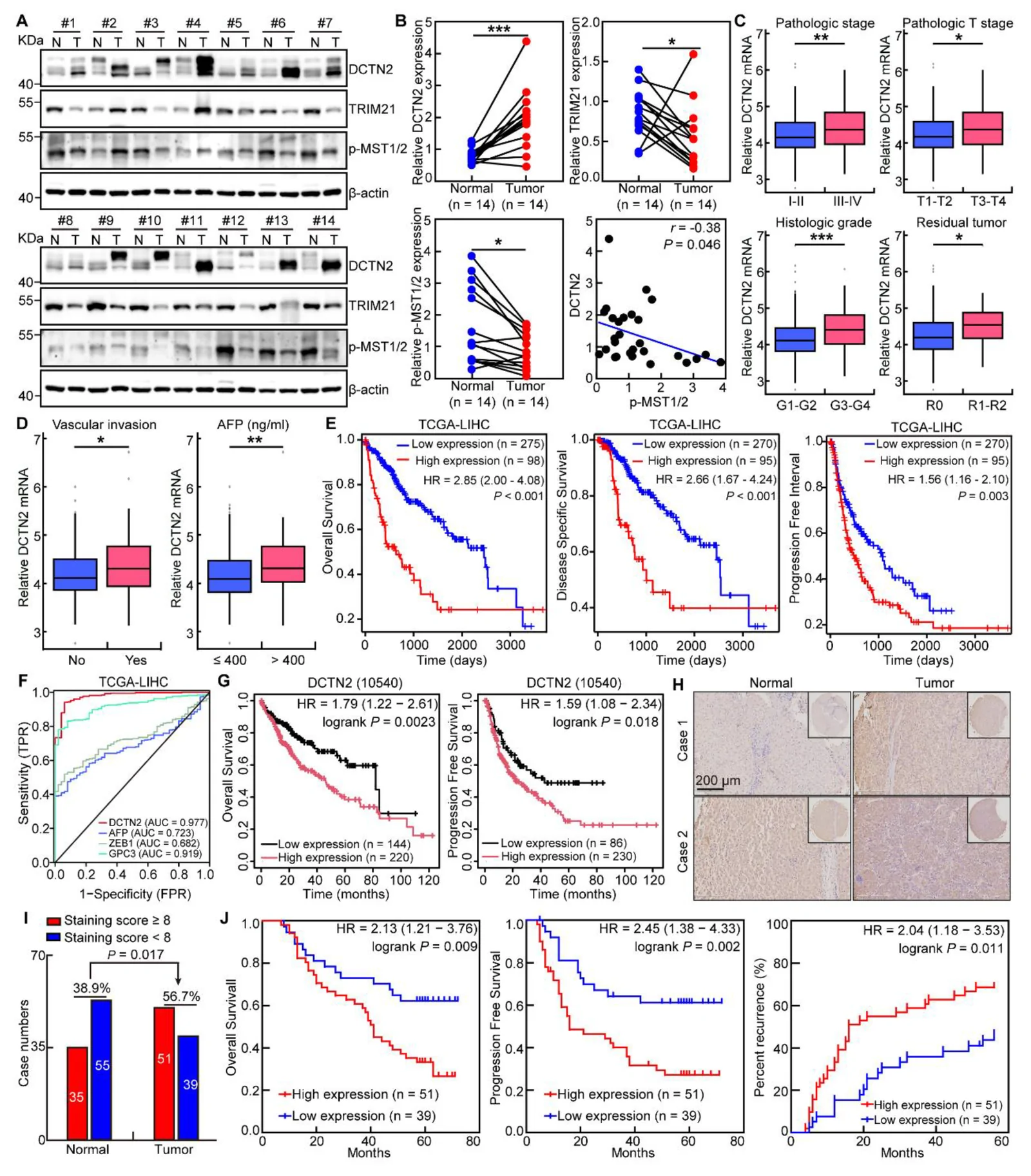
** High DCTN2 expression predicted poor clinical outcome for HCC patients. (A)** Western blot analysis showed upregulation of DCTN2 and downregulation of TRIM21 and p-MST1/2 in HCC tumor tissues compared with adjacent normal tissues. N: normal tissue; T: tumor tissue. **(B)** was the statistical chart of the results shown in (A), and the correlation between DCTN2 and p-MST1/2 was negative. **(C)** The DCTN2 mRNA expression level was significantly elevated in samples with high pathological stage, high T stage, high histological grade, and the presence of postoperative residual tumor. **(D)** High expression of DCTN2 was associated with vascular invasion and high AFP levels. **(E)** Elevated DCTN2 expression served as an independent prognostic factor for poor survival.** (F)** Receiver operating characteristic (ROC) curves for DCTN2, AFP, ZEB1 and GPC3 examined by TCGA HCC cohort. The area under the curve (AUC) numbers were shown. **(G)** HCC patients with high DCTN2 expression exhibited significantly poorer survival compared with low-expression subgroups, according to data retrieved from the KM Plotter database. **(H-I)** Representative IHC micrographs displayed DCTN2 staining in HCC tissue microarray. Quantitative analysis of staining intensity was presented in (I). **(J)** High DCTN2 expression exhibited a negative correlation with survival rate based on tissue microarray dataset. Scale bar = 200 μm. Bars indicate mean ± SD. * P < 0.05, ** P < 0.01, *** P < 0.001.

**Figure 8:**
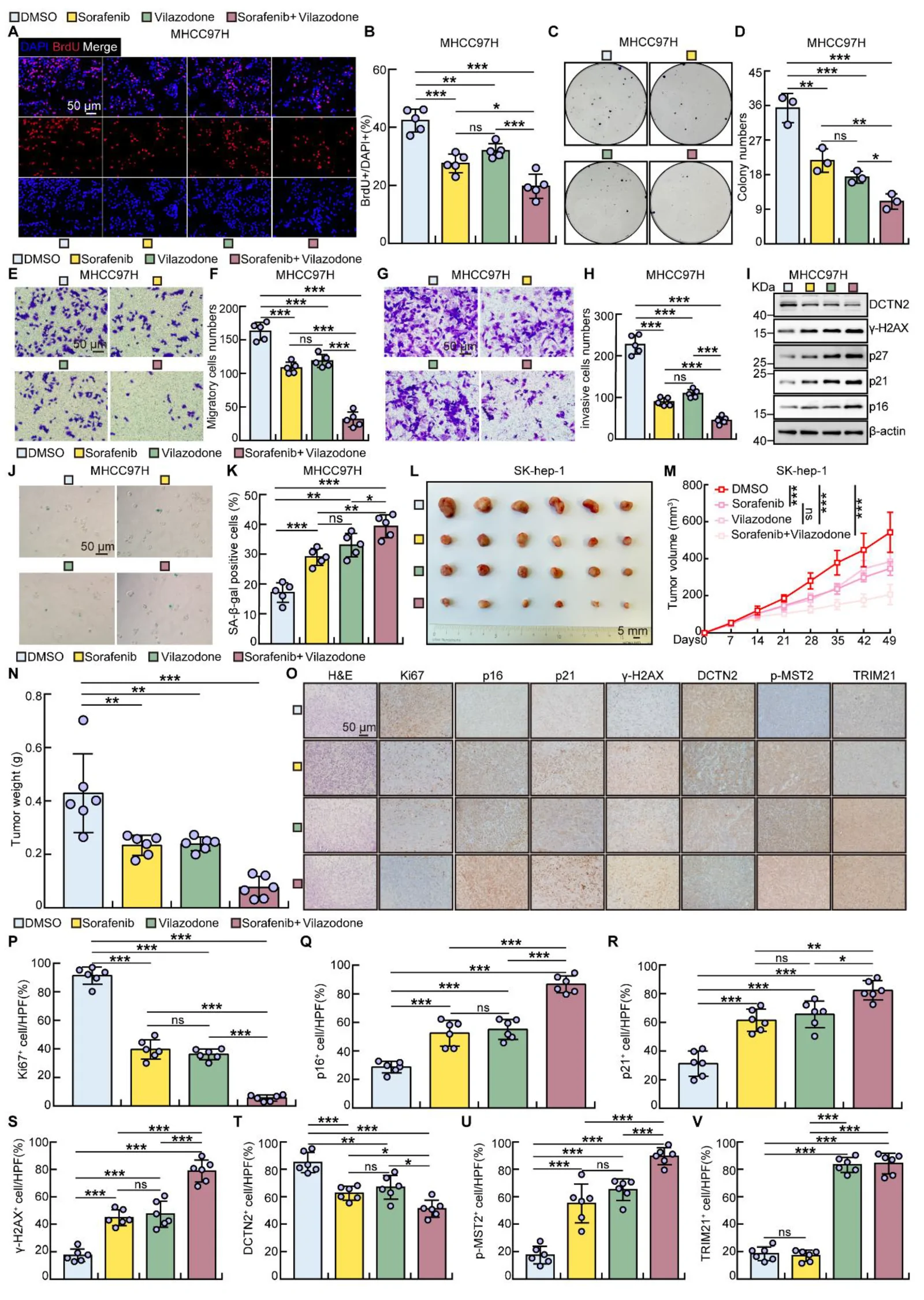
** TRIM21 activator vilazodone synergized therapeutic effect of sorafenib for HCC treatment. (A-D)** The combination of vilazodone and sorafenib additively inhibited MHCC97H cell proliferation, as evaluated by BrdU incorporation assay (A) and colony formation experiments (C). (B) and (D) were the statistical graphs of (A) and (C) respectively. Scale bar = 50 μm. **(E-H)** Vilazodone combined with sorafenib could significantly reduce the migration ability of MHCC97H cells, validated by trans-well assays (E), and invasion assays (G). (F) and (H) were the statistical graphs of (E) and (G) respectively. **(I)** The expression levels of DCTN2, γ-H2AX, p27, p21 and p16 proteins in MHCC97H cells treated with vilazodone and sorafenib were detected by western blot. **(J-K)** The combination of vilazodone and sorafenib additively induced MHCC97H cells senescence. (K) were the statistical graphs of (J). Scale bar = 50 μm. **(L-N)** In SK-hep-1 xenografts nude mice, vilazodone combined with sorafenib markedly inhibited tumor growth (M) and reduced tumor weight (N). **(O)** IHC staining of H&E, Ki67, p16, p21, γ-H2AX, DCTN2, p-MST2 and TRIM21 in xenograft tumor sections. **(P-V)** Quantitative analysis of the positive staining rates of (O) in xenograft tumor tissues (n = 6). Bars indicate mean ± SD. ns, no significant difference. * *P* < 0.05, ** *P* < 0.01, *** *P* < 0.001.

## Data Availability

Data supporting the results of this study may be obtained from the corresponding author upon reasonable request.

## Abbreviations

TP53: tumor protein p53
HCC: hepatocellular carcinoma
SA-β-gal: senescence-associated β-galactosidase
SASP: senescence-associated secretory phenotype
LATS1/2: large tumor suppressor kinase 1/2
YAP: Yes-associated protein
CDK6: cyclin D-dependent kinase 6
DCTN2: dynactin subunit 2
ATCC: American Type Culture Collection
FBS: fetal bovine serum
H&E: hematoxylin-eosin staining
IHC: immunohistochemistry
CM: conditioned medium
AFP: alpha-Fetoprotein
DSS: disease-specific survival
PFI: progression free interval
ROC: receiver operating characteristic curve
PFS: progression free survival
SREBF1: sterol regulatory element binding transcription factor 1
RETREG1: reticulophagy regulator 1
FASN: fatty acid synthase
NCL: nucleolin
RIPK1: receptor interacting serine/threonine kinase 1
ApoE: apolipoprotein E
VPS72: vacuolar protein sorting 72 homologue
TPM: transcripts per million
DEGs: differentially expressed genes
BrdU: 5-bromodeoxyuridine
EMT: epithelial-mesenchymal transition
GO: Gene Ontology
GSEA: gene set enrichment analysis
KEGG: Kyoto Encyclopedia of Genes and Genomes
OS: overall survival
PFA: paraformaldehyde
RT-qPCR: quantitative real-time PCR
TMA: tissue microarray
p-MST1/2: phospho-MST1/2
Co-IP: co-immunoprecipitation
TRIM21: tripartite motif-containing 21
IP-MS: immunoprecipitation-mass spectrometry
HUVECs: human umbilical vein endothelial cells
ChIP: chromatin immunoprecipitation
GEO: Gene Expression Omnibus
SEM: standard error of the mean
SD: standard deviation
DAPI: 4’,6-diamidino-2-phenylindole
TBST: TBS buffer containing 0.1% Tween-20
PI: propidium iodide
