# Supplementary material for: DCTN2 inhibited cellular senescence through targeting TRIM21 mediated MST2 ubiquitination and activation in hepatocellular carcinoma
Source: Theranostics. 2026 Sep 3;16(16):9165–84. doi: 10.7150/thno.137790 (PMC13684392; doi:10.7150/thno.137790)
Supplement: Supplementary file 1 [file thnov16p9165s1.pdf]

Data S1

DCTN2 inhibited cellular senescence through targeting TRIM21 mediated MST2 ubiquitination and activation in hepatocellular carcinoma

Yanbo Wang [1,2, †], Yaomei He [4, †], Qin Yu [7, †], Ningrui Wang [6, †], Qiaoling Ruan [2], Haiyan Wang [2], Hanqing Zhao [2], Yonglin Zhang [2], Yang Ke [8], Caie Tang [2], Liqi Lang [2], Yuxia Tang [2], Lixia Liu [2], Hao Zhang [2], Shan Yan [2, *], Aimin Wang [1, 5, *], Qiushuo Shen [2, *], Ceshi Chen [2, 3 *]

[†] These authors contributed equally to this work.

[*] Correspondence to: Ceshi Chen: chenc@kmmu.edu.cn

Qiushuo Shen: shenqiushuo@kmmu.edu.cn

Aimin Wang: wam@qdu.edu.cn

Shan Yan: yanshan@kmmu.edu.cn

This file includes:

Figures S1 to S7

Tables S1 to S3

Supplementary Figures

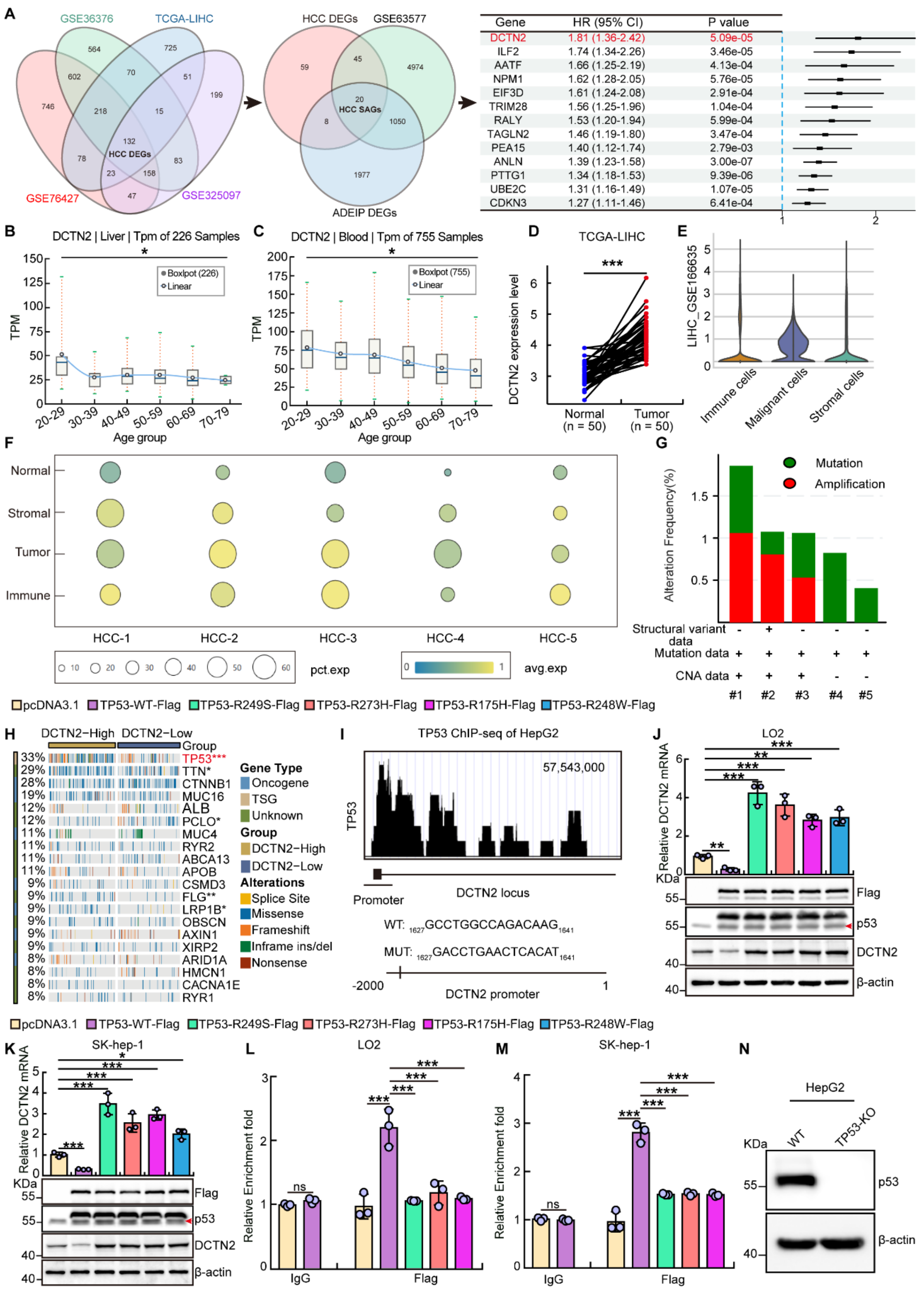

Figure S1. Senescence-related gene DCTN2 was up-regulated by TP53 mutation in HCC. (A)

Identification of 20 core HCC senescence-associated genes via Venn diagram intersection of multi-dataset DEGs including GSE76427 (|LogFC| > 0.58, P < 1E-10), GSE36376 (|LogFC| > 0.58, P < 1E-10), GSE325097 (|LogFC| > 1, P < 0.05), TCGA-LIHC GSE63577 (P < 0.05) and ADEIP DEGs (P < 0.05), accompanying with Cox proportional hazards model analysis for these 20 genes in TCGA-LIHC dataset. **(B-C)** The mean *DCTN2* transcripts per million across six age groups were retrieved from the ADEIP database for both 226 liver samples and 755 blood samples derived from healthy individuals. **(D)** Expression levels of DCTN2 in paired normal and tumor tissues extracted from the TCGA-LIHC dataset. **(E)** Distribution of DCTN2 expression across distinct cell types (immune cells, malignant cells and stromal cells) in the single-cell RNA sequencing (scRNA-seq) analysis from GSE166635 dataset, visualized as a violin plot. **(F)** Cell-type-specific spatial expression of DCTN2 from five HCC samples, visualized across four cellular compartments. Dot size = proportion of expressing cells; color = average expression intensity. **(G)** Alteration frequency of DCTN2 (mutation and amplification) in diverse hepatocellular carcinoma cohorts from online cBioPortal repository, indicating CNA, mutation, and structural variant data availability. The datasets were denoted as #5: HCC (CLCA, Nature 2024), #4: HCC (INSERM, Nat Genet 2015), #3: HCC (TCGA GDC, 2025), #2: LIHC (TCGA, PanCancer Atlas), #1: LIHC (TCGA, Firehose Legacy). **(H)** Waterfall plot showed the mutation distribution of the top 20 most frequently mutated genes in TCGA-LIHC cohort from CAMOIP database, grouped by DCTN2 expression status. **(I)** The DCTN2 promoter contained a predicted TP53 binding site identified by ChIP-seq. Data were obtained from the ENCODE project. **(J-K)** The relative expression levels of DCTN2 were detected via RT-qPCR (top) and immunoblotting (bottom) in LO2 (J) and SK-hep-1 (K) cells transfected with pcDNA3.1, TP53-WT-Flag, TP53-R249S-Flag, TP53-R175H-Flag, TP53-R248W-Flag, and TP53-R273H-Flag. The red arrow marked

the endogenous p53 protein band. **(L-M)** CUT&Tag and qPCR assays showed the enrichment of Flag-tagged TP53 and mutants at the DCTN2 promoter in LO2 (L) and SK-hep-1 (M) cells, with IgG serving as a negative control. **(N)** Immunoblotting was applied to assess TP53 knockout efficacy in HepG2 cells. Bars indicate mean ± SD. ns, no significant difference. * $P < 0.05$, ** $P < 0.01$, *** $P < 0.001$.

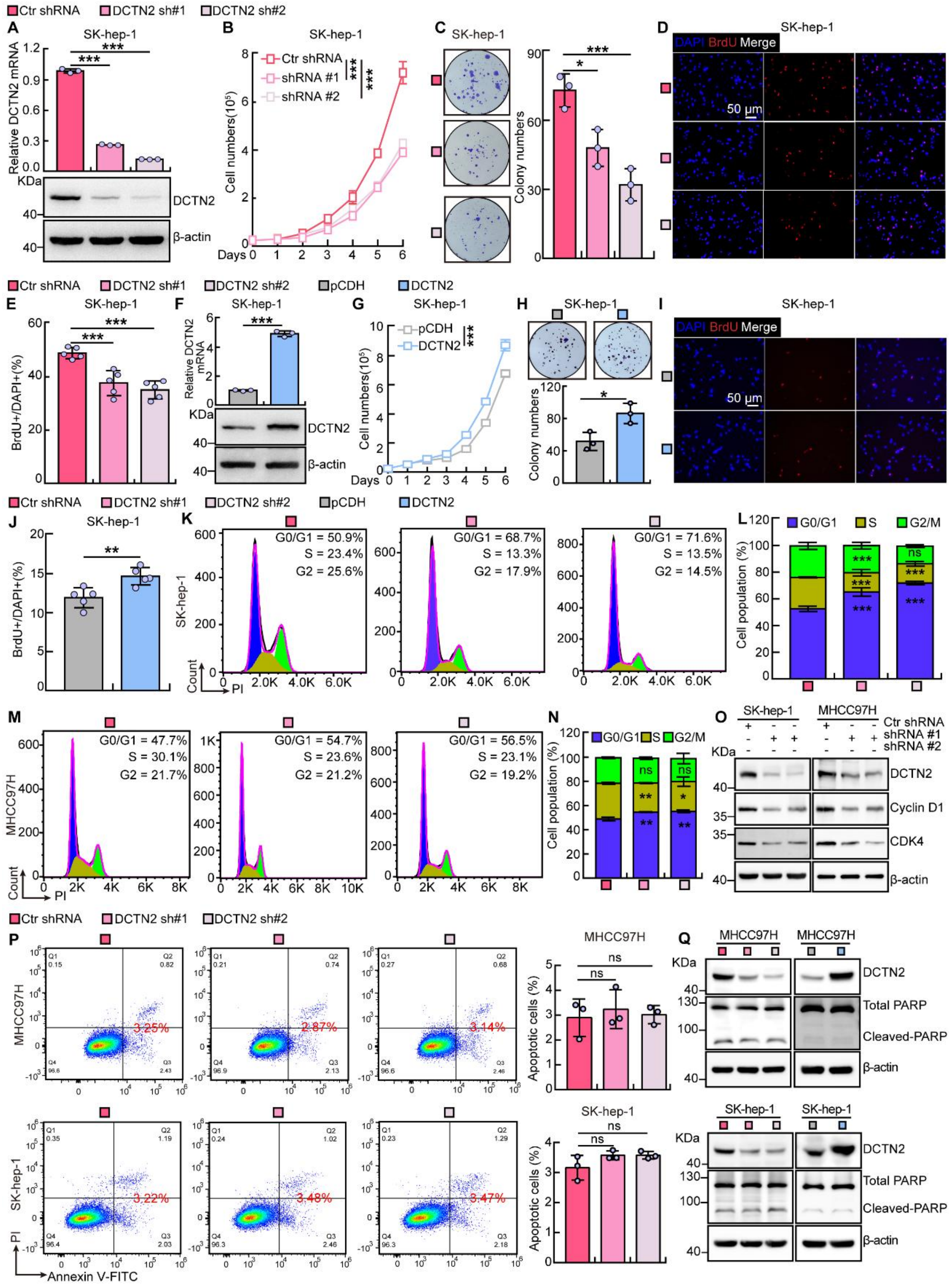

Figure S2. DCTN2 promoted tumor cell growth. (**A**) Establishment of DCTN2 knockdown in SK-hep-1 cells, verified by RT-qPCR (top) and immunoblot (bottom). (**B**) The indicated cell growth was examined

by daily counting. (**C**) DCTN2 knockdown reduced the colony formation ability of SK-hep-1 cells. (**D-E**) DCTN2 knockdown reduced BrdU positive staining cells determined by the BrdU incorporation assay in SK-hep-1 cells. (E) Quantification data for (D). Scale bar = 50 μm. (**F**) Establishment of DCTN2 overexpression in SK-hep-1 cells, verified by RT-qPCR (top) and immunoblot (bottom). (**G**) The indicated cell growth was examined by daily counting. (**H**) DCTN2 overexpression elevated the colony formation ability of SK-hep-1 cells. (**I-J**) In SK-hep-1 cells, BrdU incorporation assay showed DCTN2 overexpression increased BrdU-positive cells. (J) Quantification data for (I). Scale bar = 50 μm. (**K-N**) DCTN2 knockdown increased G0/G1 phase cell populations as examined by FACS analysis. (L, N) Quantitative data corresponding to (K, M) respectively. (**O**) Depletion of DCTN2 decreased CyclinD1 and CDK4 expression. (**P**) Annexin V/PI flow cytometry quantification of apoptotic MHCC97H and SK-hep-1 cells with DCTN2 knockdown. (**Q**) Immunoblotting for DCTN2, total/cleaved PARP in DCTN2 silenced HCC cells. Bars indicate mean ± SD. ns, no significant difference. * $P < 0.05$, ** $P < 0.01$, *** $P < 0.001$.

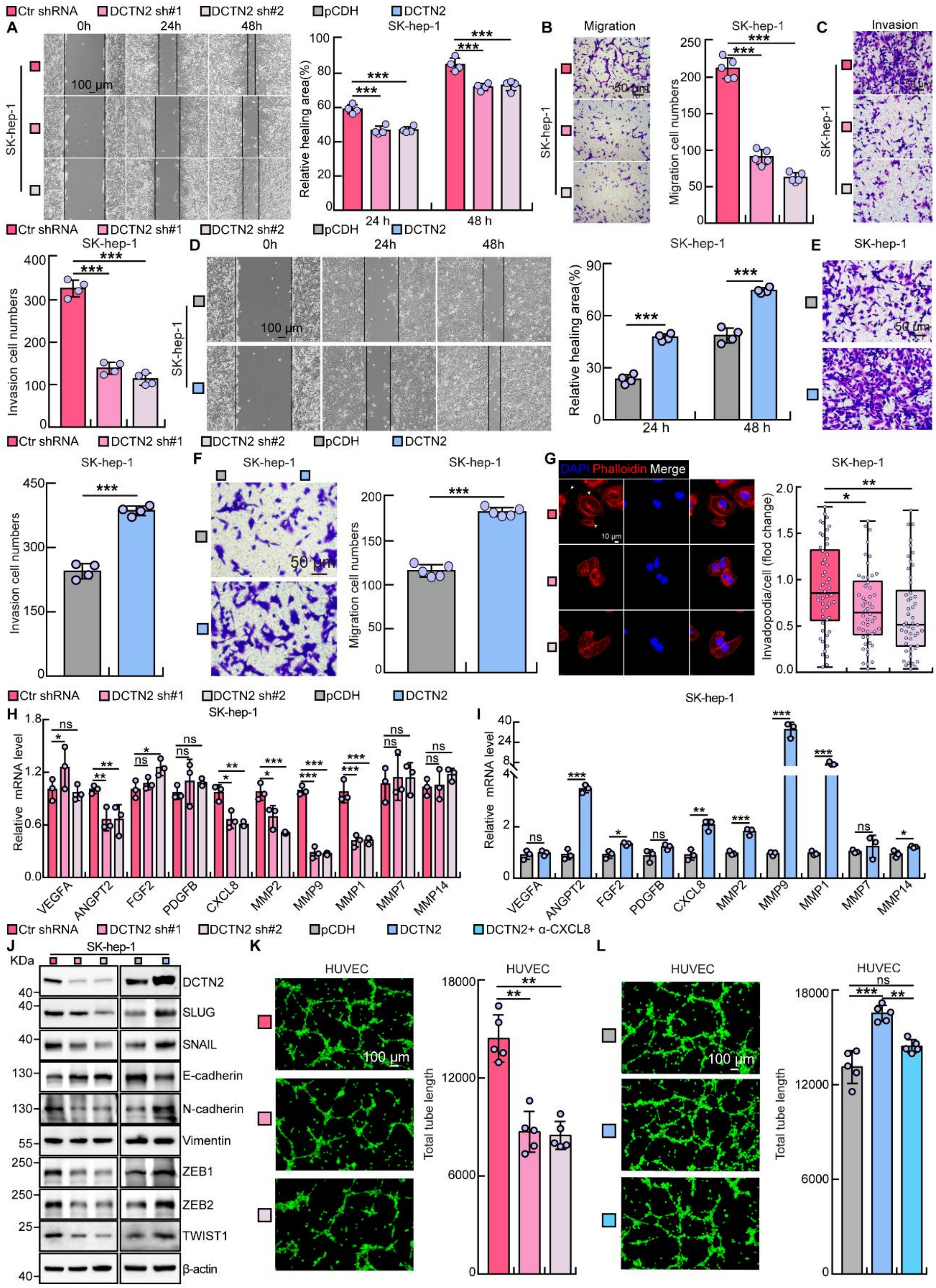

Figure S3. Ablation of DCTN2 suppressed tumor cell migration. (A-C) Wound healing (A), trans-well (C), and invasion (E) assays were conducted for evaluating how DCTN2 knockdown affected the migration

ability of SK-hep-1 cells, and quantification data were displayed in the panel on the right. Scale bars = 100 μm (A), 50 μm (B, C). **(D-F)** Wound healing (D), invasion (E), and trans-well (F) assays were used to detect the migratory ability of SK-hep-1 cells with DCTN2 overexpression. **(G)** The indicated cells underwent IF staining with phalloidin, and white head arrows highlighted structures that took on a pseudopodia-like appearance. Scale bar = 10 μm. (**H-I**) RT-qPCR quantification of pro-angiogenic mediators in SK-hep-1 cells with DCTN2 knockdown (H) or overexpression (I). (**J**) The protein expressions of SLUG, SNAIL, E-cadherin, N-cadherin, Vimentin, ZEB1, ZEB2 and TWIST1 in SK-hep-1 knockdown or overexpression cell extracts. **(K)** The capacity of primary HUVECs to form tubes was inhibited when treated with CM collected from SK-hep-1 cells with DCTN2 knockdown. Scale bar = 100 μm. (**L**) Ectopic DCTN2 overexpression promoted HUVEC angiogenesis, whereas neutralizing CXCL8 (3 μg/mL) partially abrogated this phenotype. Scale bar = 100 μm. Bars indicate mean ± SD. ns, no significant difference. * $P < 0.05$, ** $P < 0.01$, *** $P < 0.001$.

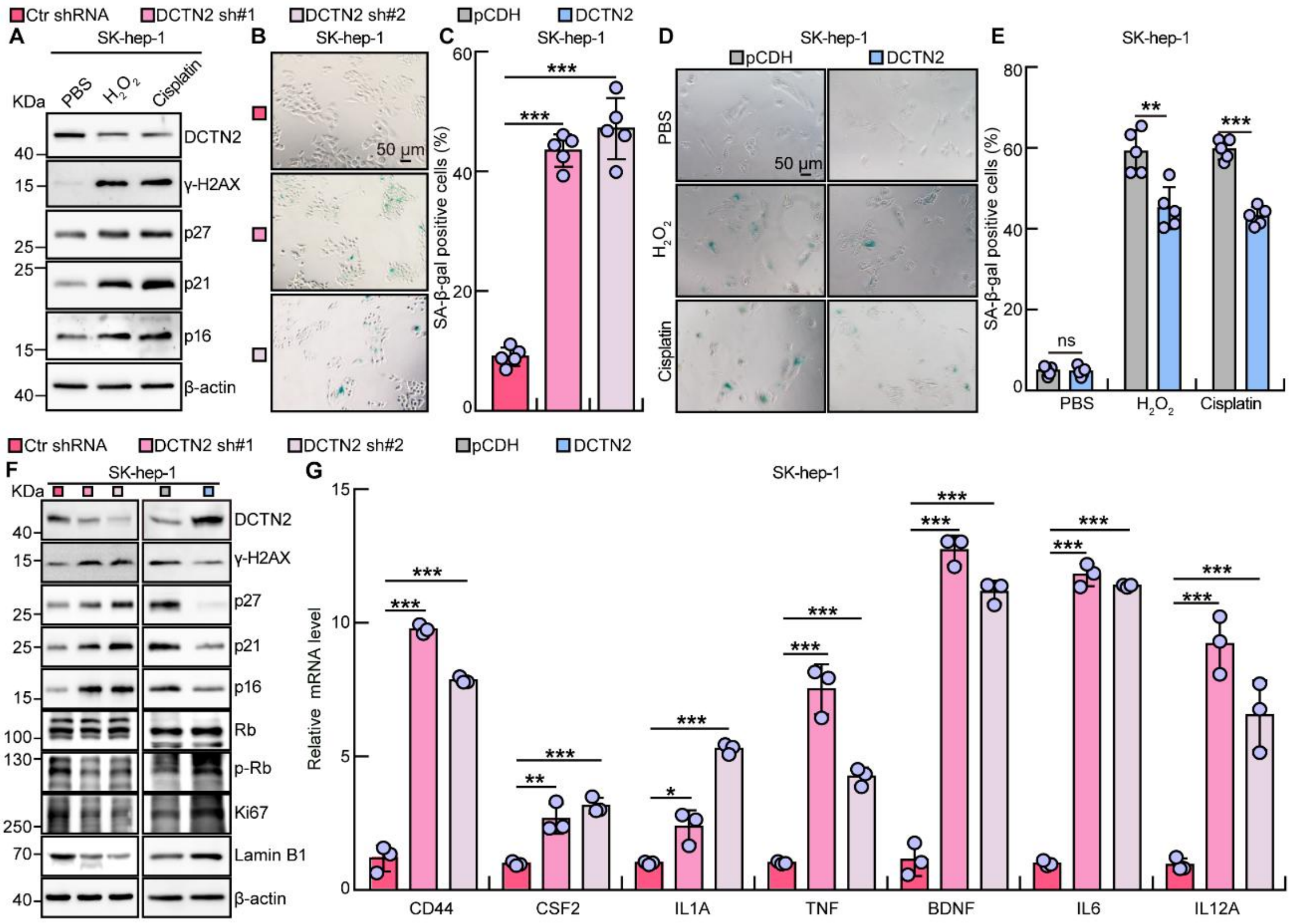

Figure S4. Inhibition of DCTN2 enhanced cellular senescence. (A) Western blot examined DCTN2, γ-H2AX, p27, p21 and p16 protein levels in H_2O_2 or cisplatin treated SK-hep-1 cells. **(B-E)** SA-β-gal activity changes in DCTN2-knockdown or overexpressing SK-hep-1 cells; (C) and (E) were the statistical graphs of (B) and (D), respectively. Scale bar = 50 μm. Data represented the percentage of SA-β-gal-positive cells ± SD. **(F)** Immunoblotting examined DCTN2, γ-H2AX, p27, p21, p16, Rb, p-Rb, Ki67 and Lamin B1 protein expression in SK-hep-1 cells with DCTN2 depletion or overexpression. (**G**) RT-qPCR examined the mRNA expression levels of SASP including CD44, CSF2, interleukin 1A, 6, 12A, TNF and BDNF in DCTN2-depleted SK-hep-1 cells. Bars indicate mean ± SD. ns, no significant difference. * $P < 0.05$, ** $P < 0.01$, *** $P < 0.001$.

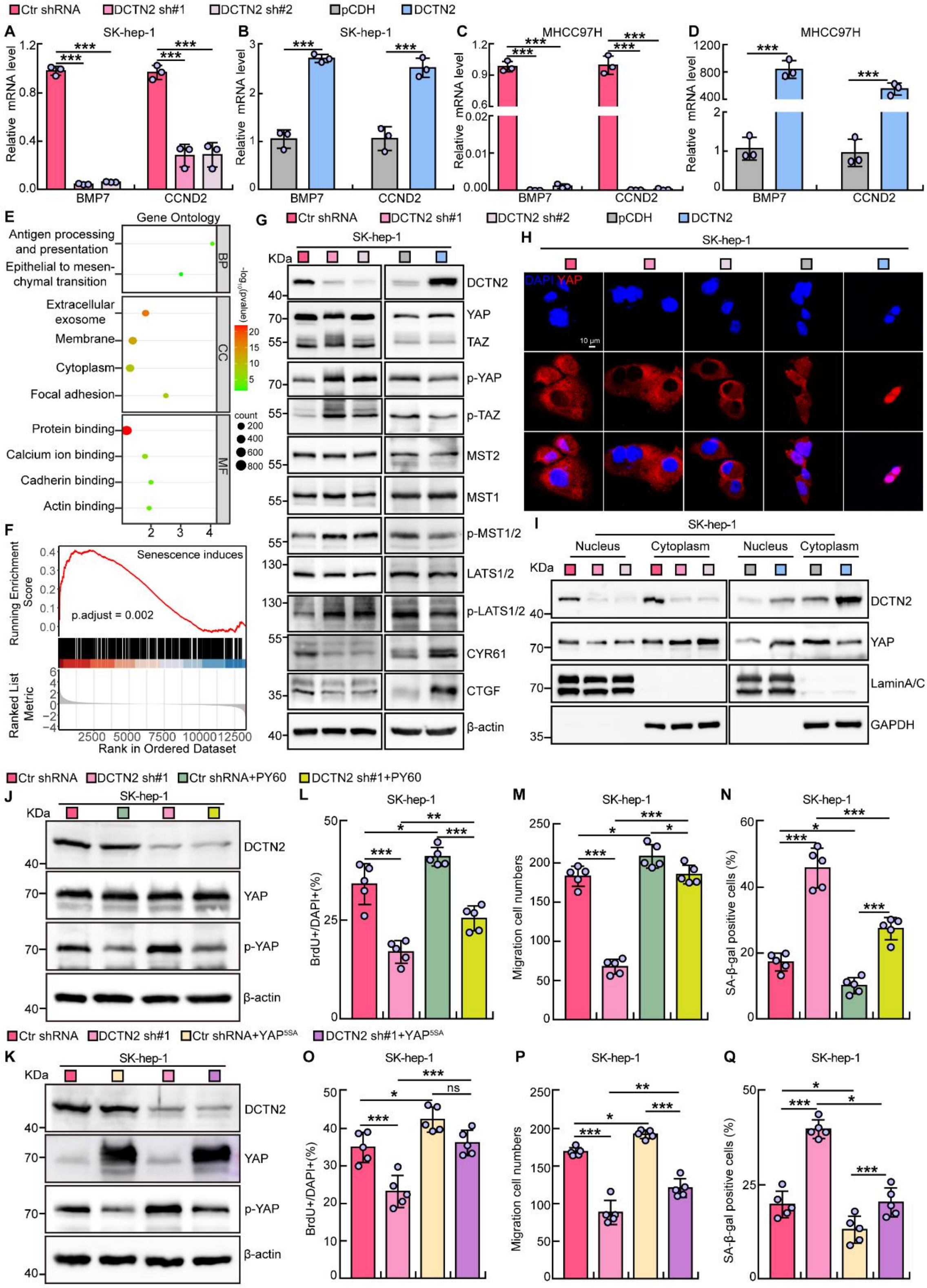

Figure S5. DCTN2 restrained the Hippo signaling pathway. (A-D) RT-qPCR was performed to validate BMP7 and CCND2, the downstream genes of DCTN2 identified from RNA-seq results. **(E)** GO enrichment

analysis of DEGs identified relevant pathways perturbed by DCTN2 depletion. **(F)** GSEA analysis from RNA-seq data indicated the involvement of DCTN2 in HCC cellular senescence. **(G)** Western blot was used to detect the protein levels of key Hippo signaling pathway components and its downstream target genes CYR61, CTGF in SK-hep-1 cells under DCTN2 overexpression or knockdown. **(H-I)** Immunofluorescence staining (H) and western blot of nucleus-cytoplasm fractionation (I) were utilized to determine the subcellular localization of YAP in SK-hep-1 cells. Scale bar = 10 μm. **(J-K)** Immunoblotting was employed to validate the protein levels of DCTN2, YAP and p-YAP in indicated SK-hep-1 cells. **(L-Q)** Treatment with PY60 or ectopic expression of YAP^{5SA} was able to restore the phenotypes in DCTN2-deleted SK-hep-1 cells. Bars indicate mean ± SD. ns, no significant difference. * $P < 0.05$, ** $P < 0.01$, *** $P < 0.001$.

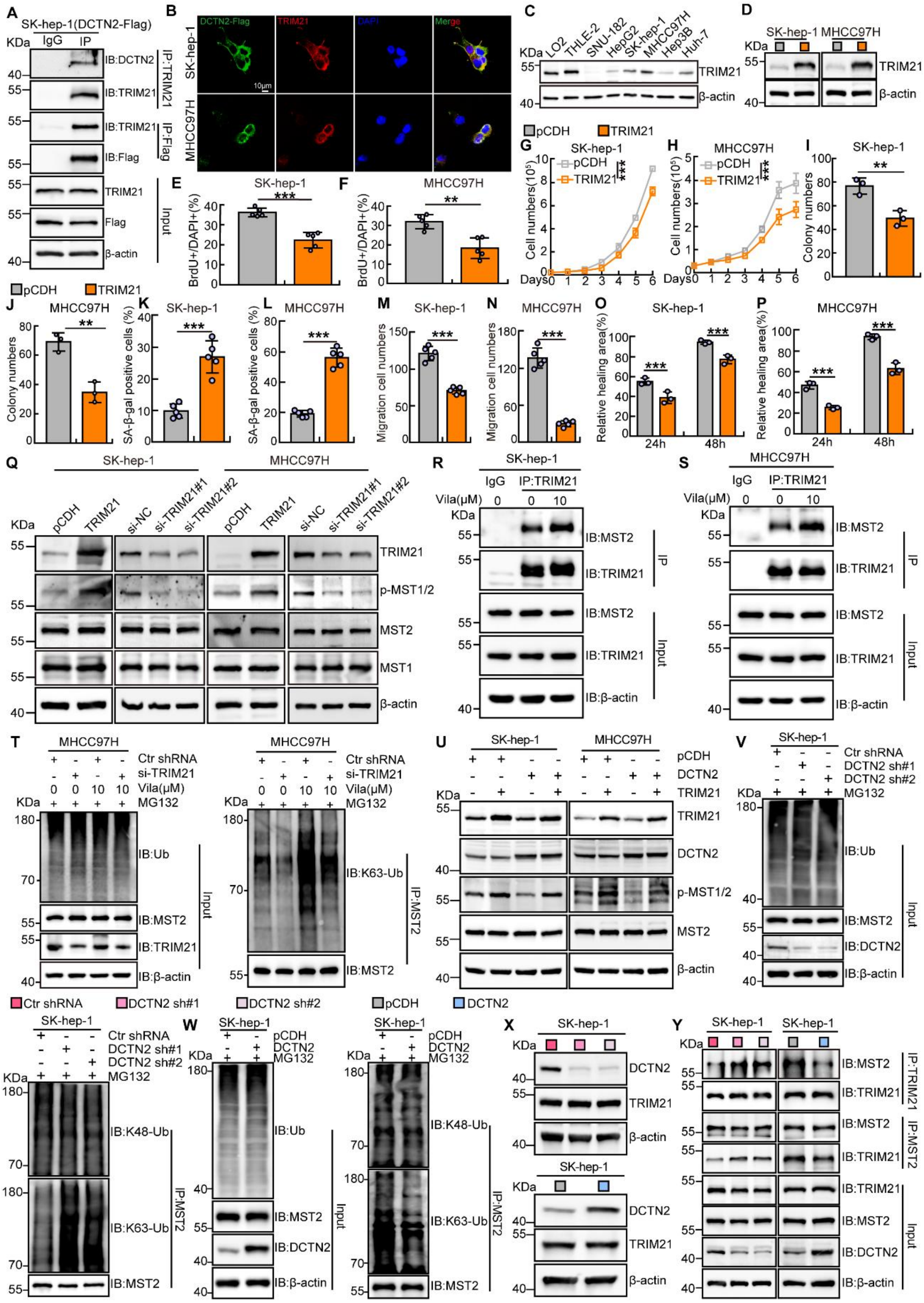

Figure S6. TRIM21 suppressed HCC malignancy. (A) Co-IP assay using Flag and TRIM21 antibodies in DCTN2-Flag overexpressing SK-hep-1 cells. IgG was used as a negative control. **(B)**

Immunofluorescence assay indicated the co-localization of DCTN2 with TRIM21 in SK-hep-1 and MHCC97H cells. **(C)** The relative expression level of TRIM21 in HCC cell lines including LO2, THLE-2, SNU-182, HepG2, SK-hep-1, MHCC97H, Hep3B and Huh7 examined by immunoblotting assay. **(D)** TRIM21-overexpressing SK-Hep-1 and MHCC97H cell lines were established and verified via western blot. **(E-J)** TRIM21 overexpression decreased SK-hep-1 and MHCC97H cells' proliferation abilities by BrdU incorporation (E, F), growth curve (G, H), and colony formation experiments (I, J). (**K-L**) β-gal staining quantification result of SK-hep-1 (K) and MHCC97H (L) cells with stable overexpression of DCTN2. **(M-P)** Forced expression of TRIM21 inhibited cell migration in trans-well assays (M, N) and wound healing (O, P). **(Q)** The protein expressions of TRIM21, p-MST1/2, MST2, and MST1 were detected by immunoblot with the indicated antibodies in SK-hep-1 and MHCC97H cell extracts. **(R-S)** Co-IP analysis demonstrating that vilazodone enhanced endogenous TRIM21-MST2 interaction in SK-hep-1 (R) and MHCC97H (S) HCC cells. Cells were exposed to 0 or 10 μM vilazodone. IgG served as negative control for immunoprecipitation. **(T)** Ubiquitination analysis of MST2 in MHCC97H cells with TRIM21 knockdown and vilazodone intervention. **(U)** Western blot analysis of TRIM21, DCTN2, p-MST1/2 and MST2 expression in SK-hep-1 and MHCC97H cells transfected with or without DCTN2-expressing plasmid and TRIM21-expressing plasmid. **(V-W)** DCTN2 mainly mediated K63-linked polyubiquitination of MST2. SK-hep-1 cells with DCTN2 knockdown (V) or overexpression (W) were subjected to anti-MST2 immunoprecipitation, followed by *in vivo* ubiquitination assay to detect K48- and K63-linked polyubiquitination of MST2 using chain-specific antibodies. **(X)** Western blot was used to assess TRIM21 expression in MHCC97H cells with DCTN2 silencing or overexpression. **(Y)** Co-IP assay was performed to investigate the interaction between DCTN2 and TRIM21 in SK-hep-1 cells with DCTN2 overexpression

or knockdown. Bars indicate mean ± SD. * $P < 0.05$, ** $P < 0.01$, *** $P < 0.001$.

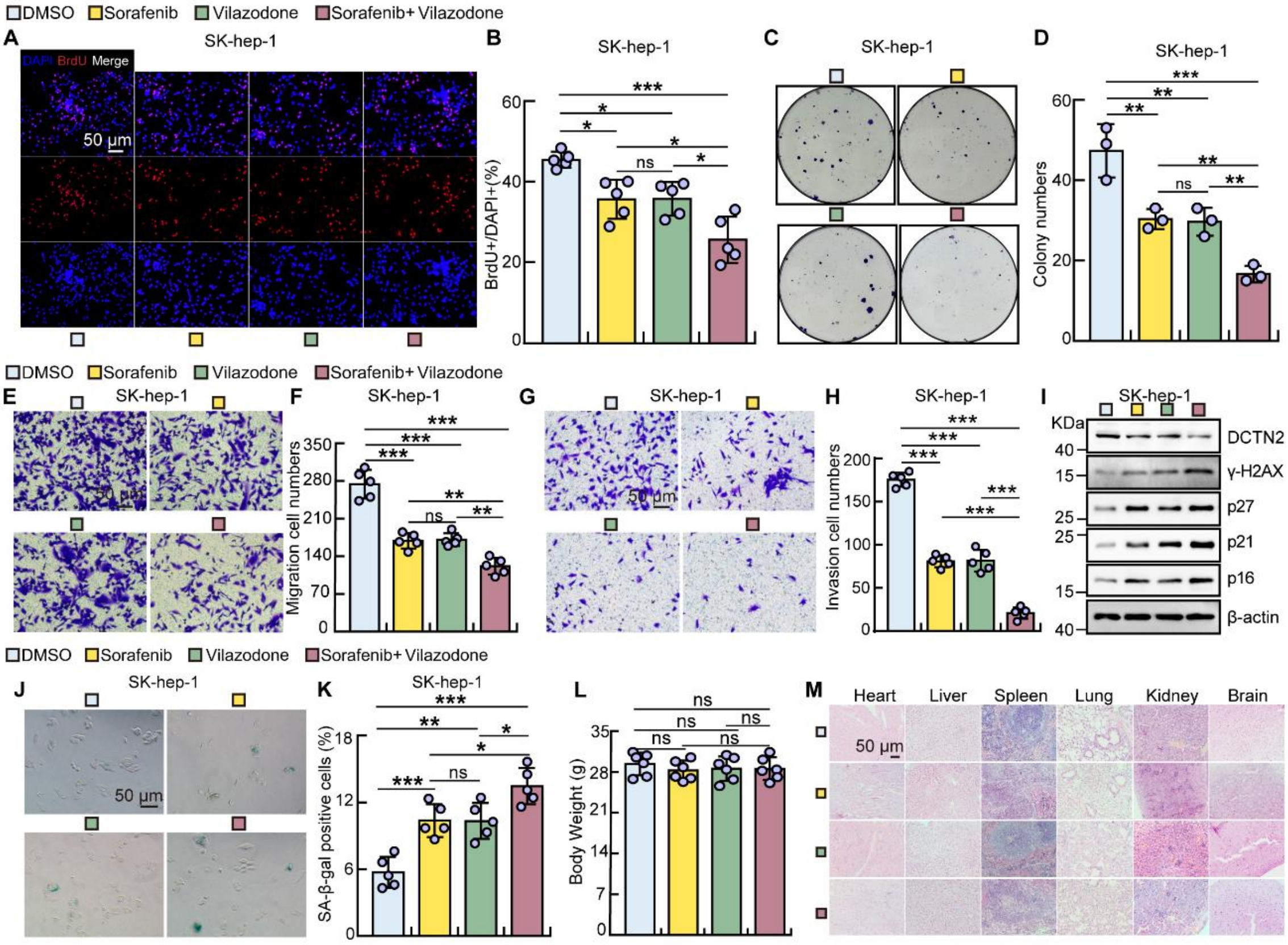

Figure S7. The TRIM21 activator vilazodone exhibited an additive therapeutic effect in combination with sorafenib. (A-D) The co-treatment with vilazodone and sorafenib led to an additive inhibition of SK-hep-1 cell proliferation, as evidenced by the BrdU incorporation assay (A) and colony formation assay (C). The statistical analyses for (A) and (C) were presented in (B) and (D), respectively. **(E-H)** The combination of vilazodone and sorafenib notably diminished the migration capacity of SK-hep-1 cells, as confirmed by trans-well assays (E) and invasion assays (G). The statistical data for (E) and (G) were presented in (F) and (H), respectively. **(I)** Western blot analysis was performed to assess the expression levels of DCTN2, γ-H2AX, p27, p21, and p16 proteins in SK-hep-1 cells treated with vilazodone and sorafenib. **(J-K)** The co-treatment with vilazodone and sorafenib resulted in an obvious induction of SK-hep-1 cell senescence, with

statistical data shown in (K) corresponding to (J). Scale bar = 50 μm. **(L)** There were no significant changes in the body weight of nude mice by treatment of SK-hep-1 with vilazodone in combination with sorafenib (n = 6 per group). **(M)** The heart, liver, spleen, lung, brain and kidney of nude mice were harvested, followed by H&E staining to assess potential drug toxicity. No noticeable morphological alterations were observed in the organs post-treatment with the drug. Scale bar = 50 μm. Bars indicate mean ± SD. ns, no significant difference. * $P < 0.05$, ** $P < 0.01$, *** $P < 0.001$.

Supplementary Tables

Table S1. Antibodies and oligos used in this study.

Antibody Name	Catalog number	Supplier	Species
p53	sc-126	Santa Cruz	Mouse
DCTN2	A2200	abclonal	Rabbit
p16	23200S	CST	Rabbit
β-actin	3700S	CST	Mouse
p21	2947S	CST	Rabbit
p27	3686S	CST	Rabbit
Ki67	RMA-0731	MXB	Rabbit
p-MST2	bs4634R	Bioss	Rabbit
p-MST1/2	AP1564	abclonal	Rabbit
CyclinD1	55506	CST	Rabbit
CDK4	553048	Biosciences	Mouse
γ-H2A.X	7631S	CST	Rabbit
YAP/TAZ	8418S	CST	Rabbit
p-YAP	4911S	CST	Rabbit
p-TAZ	AF4315	Affinity	Rabbit
MST1	22245-1-AP	proteintech	Rabbit
Snail	13099-1-AP	proteintech	Rabbit

Slug	12129-1-AP	proteintech	Rabbit
MST2	A9036	abclonal	Rabbit
MST2	PA5-29320	thermo	Mouse
LATS1/2	DF7517	Affinity	Rabbit
p-LATS1/2	AF8163	Affinity	Rabbit
K48-Ub	A3606	abclonal	Rabbit
K63-Ub	A18164	abclonal	Rabbit
CTGF	ER1802-69	HUABIO	Rabbit
CYR61	ER1905-70	HUABIO	Rabbit
YAP	14074S	CST	Rabbit
LaminA/C	81042-1-RR	proteintech	Rabbit
GAPDH	60004-1-Ig	proteintech	Mouse
Flag	14793S	CST	Rabbit
Flag	F1804	Sigma	Mouse
HA	SC-7392	Santa Cruz	Mouse
HA	3724S	CST	Rabbit
TRIM21	67136-1-Ig	proteintech	Mouse
TRIM21	DF6717	Affinity	Rabbit
Myc	2278S	CST	Rabbit
Ub	80992-1-RR	proteintech	Rabbit

RB	10048-2-Ig	proteintech	Rabbit
N-cadherin	22018-1-AP	proteintech	Rabbit
E-cadherin	20874-1-AP	proteintech	Rabbit
Vimentin	10366-1-AP	proteintech	Rabbit
PARP	9532S	CST	Rabbit
ZEB2	A5705	abclonal	Rabbit
ZEB1	EM1710-27	HUABIO	Mouse
TWIST1	25465-1-AP	Proteintech	Rabbit
P-RB	30376-1-AP	Proteintech	Rabbit
Ki-67	84192-4-RR	Proteintech	Rabbit
Lamin B1	13435S	CST	Rabbit
CXCL8	MAB208	R&D	Mouse

**Primer Name**	**Primer Sequences (5'-3')**
Human β-actin_F	AAGTGTGACGTGGACATCCGC
Human β-actin_R	CCGGACTCGTCATACTCCTGCT
Human DCTN2_F	ATGGCGGACCCTAAATACGC
Human DCTN2_R	TCAGGTAGGTCGCTAGTTTCATA
Human p53_F	CAGCACATGACGGAGGTTGT
Human p53_R	TCATCCAAATACTCCACACGC
Ctr shRNA_F	GCACTACCAGAGCTAACTCAG

Ctr shRNA_R	CTGAGTTAGCTCTGGTAGTGC
Human DCTN2 shRNA #1_F	GCCTCTGTAGAAGATGCAGAT
Human DCTN2 shRNA #1_R	ATCTGCATCTTCTACAGAGGC
Human DCTN2 shRNA #2_F	CAACTGTTTCTGACCATTAAA
Human DCTN2 shRNA #2_R	TTTAATGGTCAGAAACAGTTG
Negative Control siRNA_F	UUCUCCGAACGUGUCACGUTT
Negative Control siRNA_R	ACGUGACACGUUCGGAGAATT
Human p53 siRNA #1_F	CGGCGCACAGAGGAAGAGAAU
Human p53 siRNA #1_R	AUUCUCUUCCUCUGUGCGCCG
Human p53 siRNA #2_F	GUCCAGAUGAAGCUCCCAGAA
Human p53 siRNA #2_R	UUCUGGGAGCUUCAUCUGGAC
Human TRIM21 siRNA #1_F	GACUUCACCUGUUCUGUGATT
Human TRIM21 siRNA #1_R	UCACAGAACAGGUGAAGUCTT
Human TRIM21 siRNA #2_F	CAGCACGCUUGACAAUGAUTT
Human TRIM21 siRNA #2_R	AUCAUUGUCAAGCGUGCUGTT
Human CD44_F	GCCGCTTTGCAGGTGTATT
Human CD44_R	TCCATCTGGGCCATTGTG
Human CSF2_F	CCCTGGGAGCATGTGAAT
Human CSF2_R	GCCCTTGAGCTTGGTGAG
Human IL1A_F	GGTTGAGTTTAAGCCAATCCA

Human IL1A_R	TGCTGACCTAGGCTTGATGA
Human TNF_F	TCCAGGCGGTGCTTGTTC
Human TNF_R	GGCTACAGGCTTGTCACTCG
Human BDNF_F	CTCTTTCTGCTGGAGGAATACA
Human BDNF_R	CGCCGTTACCCACTCACT
Human IL6_F	GAAAGCAGCAAAGAGGCAC
Human IL6_R	GCTCTGGCTTGTTCCTCACTAC
Human IL12A_F	TGGCTACCCTGGTCCTCCT
HumanIL12A_R	GCCTCCACTGTGCTGGTTTTAT
Human BMP7_F	TCGGCACCCATGTTCATGC
Human BMP7_R	GAGGAAATGGCTATCTTGCAGG
Human CCND2_F	TTTGCCATGTACCCACCGTC
Human CCND2_R	AGGGCATCACAAGTGAGCG
DNA Spike in Primer F	GCCTTCTTCCCATTTCTGATCC
DNA Spike in Primer R	CACGAATCAGCGGTAAAGGT
hDCTN2 Cut&Tag qPCR primer_F	TCTGCTCAGCAACTGTCTGG
hDCTN2 Cut&Tag qPCR primer_R	TTCCCTTAAGATTCAAGGTAGGTT
Human VEGFA_F	AGGGCAGAATCATCACGAAGT
Human VEGFA_R	AGGGTCTCGATTGGATGGCA
Human ANGPT2_F	AACTTTCGGAAGAGCATGGAC

Human ANGPT2_R	CGAGTCATCGTATTCGAGCGG
Human FGF2_F	AGAAGAGCGACCCTCACATCA
Human FGF2_R	CGGTTAGCACACACTCCTTTG
Human PDGFB_F	CTCGATCCGCTCCTTTGATGA
Human PDGFB_R	CGTTGGTGCGGTCTATGAG
Human CXCL8_F	GACATACTCCAAACCTTTCCACC
Human CXCL8_R	AAACTTCTCCACAACCCTCTGC
Human MMP2_F	TACAGGATCATTGGCTACACACC
Human MMP2_R	GGTCACATCGCTCCAGACT
Human MMP9_F	TGTACCGCTATGGTTACACTCG
Human MMP9_R	GGCAGGGACAGTTGCTTCT
Human MMP1_F	AAAATTACACGCCAGATTTGCC
Human MMP1_R	GGTGTGACATTACTCCAGAGTTG
Human MMP7_F	GAGTGAGCTACAGTGGGAACA
Human MMP7_R	CTATGACGCGGGAGTTTAACAT
Human MMP14_F	GGCTACAGCAATATGGCTACC
Human MMP14_R	GATGGCCGCTGAGAGTGAC

Table S2. Bioinformatical databases used in this study.

Name	Link	Targets
ADEIP	https://gb.whu.edu.cn/ADEIP/	To investigate DCTN2 expression pattern in 226 liver and 755 blood samples across six age groups of healthy individuals.
HCCDB V2	http://lifeome.net/database/hccdb/home.html	To validate DCTN2 expression in the spatial transcriptomics data.
CAMOIP	http://www.zjyy-oncology.com:20002/	To explore the correlation between DCTN2 expression and TP53 mutation status.
GSEA	https://www.gsea_msigdb.org/gsea/index.jsp	To examine the correlation between DCTN2 and cellular senescence process.
KEGG	https://www.genome.jp/kegg/catalog/org_list.html	To examine DCTN2 regulated Hippo signaling pathway.
GO	http://geneontology.org/	To examine DCTN2 regulated Hippo signaling pathway.
Kaplan Meier Plotter	https://kmplot.com/analysis/index.php?p=background	To examine the correlation between DCTN2 and survival time of HCC patients.
ROC Plotter	https://www.rocplot.org/	To examine the correlation between DCTN2 and prognosis of HCC patients.

GEO	https://www.ncbi.nlm.nih.gov/geo/	To examine the expression of DCTN2 in different GEO datasets.
cBioPortal	http://www.cbioportal.org/	To examine the mutation and amplification status in HCC.
ENCODE	https://www.encodeproject.org/	To examine whether the DCTN2 promoter contains a predicted TP53 binding site

Table S3. Clinicopathological characteristics of liver cancer patients in this study.

Patient characteristics	Clinical parameters	Cases	Low expression	High expression	*P* value
**Gender**	Male	74	30	44	
	Female	16	9	7	0.251
**Age (years)**	< 60	68	39	29	
	≥ 60	22	16	6	0.199
**Clinical stage**	I/II	58	23	35	
	III/IV	32	16	16	0.343
**Tumor size (cm)**	< 5	44	21	23	
	≥ 5	46	18	28	0.411
**Total bilirubin (μM)**	< 17	64	30	34	
	≥ 17	26	9	17	0.287
**ALT (U/L)**	< 40	41	21	20	
	≥ 40	49	18	31	0.167
**ALB (g/dL)**	< 40	22	7	15	
	≥ 40	68	32	36	0.210
**AFP (μg/L)**	< 400	57	29	28	
	≥ 400	33	10	23	0.058
**GGT (U/L)**	< 50	35	22	13	
	≥ 50	55	17	38	**0.003**

ALT: Alanine Aminotransferase

ALB: Albumin

AFP: Alpha-fetoprotein

GGT: γ-glutamyl transpeptidase

Data S3. Full gels/blots images used in this study

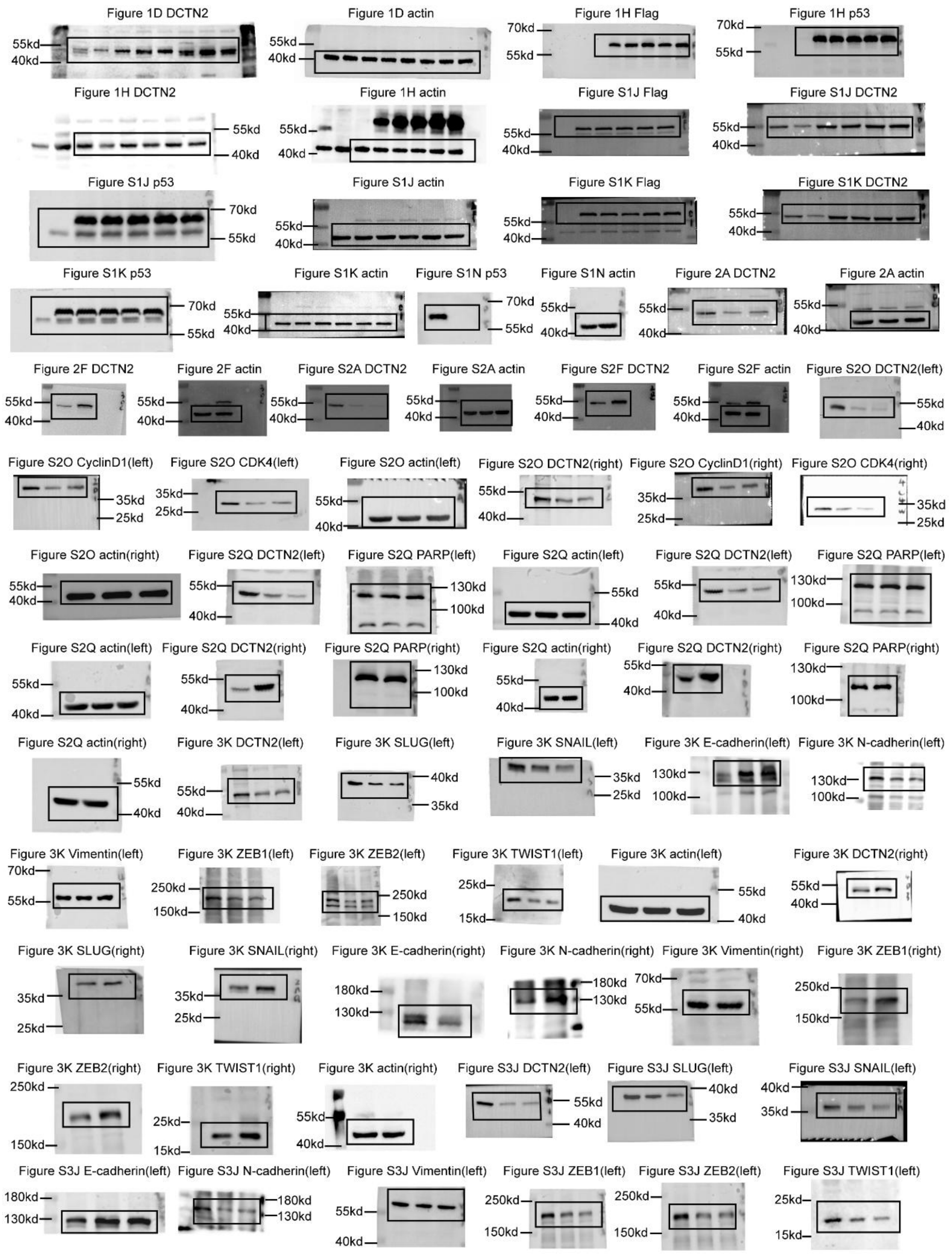

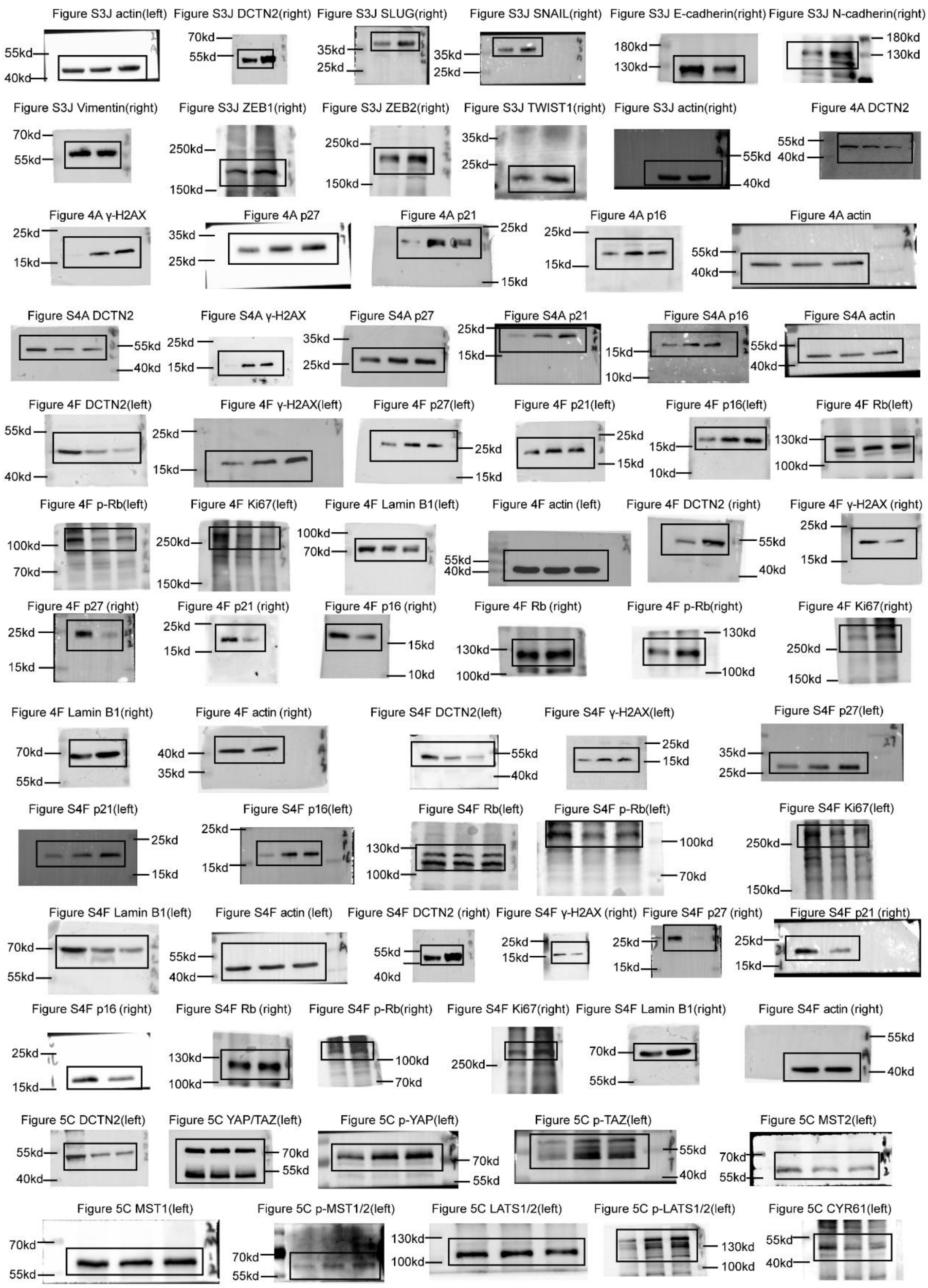

Figure S3J actin(left) 55kd 40kd
Figure S3J DCTN2(right) 70kd 55kd
Figure S3J SLUG(right) 35kd 25kd
Figure S3J SNAIL(right) 35kd 25kd
Figure S3J E-cadherin(right) 180kd 130kd
Figure S3J N-cadherin(right) 180kd 130kd
Figure S3J Vimentin(right) 70kd 55kd
Figure S3J ZEB1(right) 250kd 150kd
Figure S3J ZEB2(right) 250kd 150kd
Figure S3J TWIST1(right) 35kd 25kd
Figure S3J actin(right) 55kd 40kd
Figure 4A DCTN2 55kd 40kd
Figure 4A γ-H2AX 25kd 15kd
Figure 4A p27 35kd 25kd
Figure 4A p21 25kd 15kd
Figure 4A p16 25kd 15kd
Figure 4A actin 55kd 40kd
Figure S4A DCTN2 55kd 40kd
Figure S4A γ-H2AX 25kd 15kd
Figure S4A p27 35kd 25kd
Figure S4A p21 25kd 15kd
Figure S4A p16 15kd 10kd
Figure S4A actin 55kd 40kd
Figure 4F DCTN2(left) 55kd 40kd
Figure 4F γ-H2AX(left) 25kd 15kd
Figure 4F p27(left) 25kd 15kd
Figure 4F p21(left) 25kd 15kd
Figure 4F p16(left) 15kd 10kd
Figure 4F Rb(left) 130kd 100kd
Figure 4F p-Rb(left) 100kd 70kd
Figure 4F Ki67(left) 250kd 150kd
Figure 4F Lamin B1(left) 100kd 70kd
Figure 4F actin (left) 55kd 40kd
Figure 4F DCTN2 (right) 55kd 40kd
Figure 4F γ-H2AX (right) 25kd 15kd
Figure 4F p27 (right) 25kd 15kd
Figure 4F p21 (right) 25kd 15kd
Figure 4F p16 (right) 15kd 10kd
Figure 4F Rb (right) 130kd 100kd
Figure 4F p-Rb(right) 130kd 100kd
Figure 4F Ki67(right) 250kd 150kd
Figure 4F Lamin B1(right) 70kd 55kd
Figure 4F actin (right) 40kd 35kd
Figure S4F DCTN2(left) 55kd 40kd
Figure S4F γ-H2AX(left) 25kd 15kd
Figure S4F p27(left) 35kd 25kd
Figure S4F p21(left) 25kd 15kd
Figure S4F p16(left) 25kd 15kd
Figure S4F Rb(left) 130kd 100kd
Figure S4F p-Rb(left) 100kd 70kd
Figure S4F Ki67(left) 250kd 150kd
Figure S4F Lamin B1(left) 70kd 55kd
Figure S4F actin (left) 55kd 40kd
Figure S4F DCTN2 (right) 55kd 40kd
Figure S4F γ-H2AX (right) 25kd 15kd
Figure S4F p27 (right) 25kd 15kd
Figure S4F p21 (right) 25kd 15kd
Figure S4F p16 (right) 25kd 15kd
Figure S4F Rb (right) 130kd 100kd
Figure S4F p-Rb(right) 100kd 70kd
Figure S4F Ki67(right) 250kd
Figure S4F Lamin B1(right) 70kd 55kd
Figure S4F actin (right) 55kd 40kd
Figure 5C DCTN2(left) 55kd 40kd
Figure 5C YAP/TAZ(left) 70kd 55kd
Figure 5C p-YAP(left) 70kd 55kd
Figure 5C p-TAZ(left) 55kd 40kd
Figure 5C MST2(left) 70kd 55kd
Figure 5C MST1(left) 70kd 55kd
Figure 5C p-MST1/2(left) 70kd 55kd
Figure 5C LATS1/2(left) 130kd 100kd
Figure 5C p-LATS1/2(left) 130kd 100kd
Figure 5C CYR61(left) 55kd 40kd

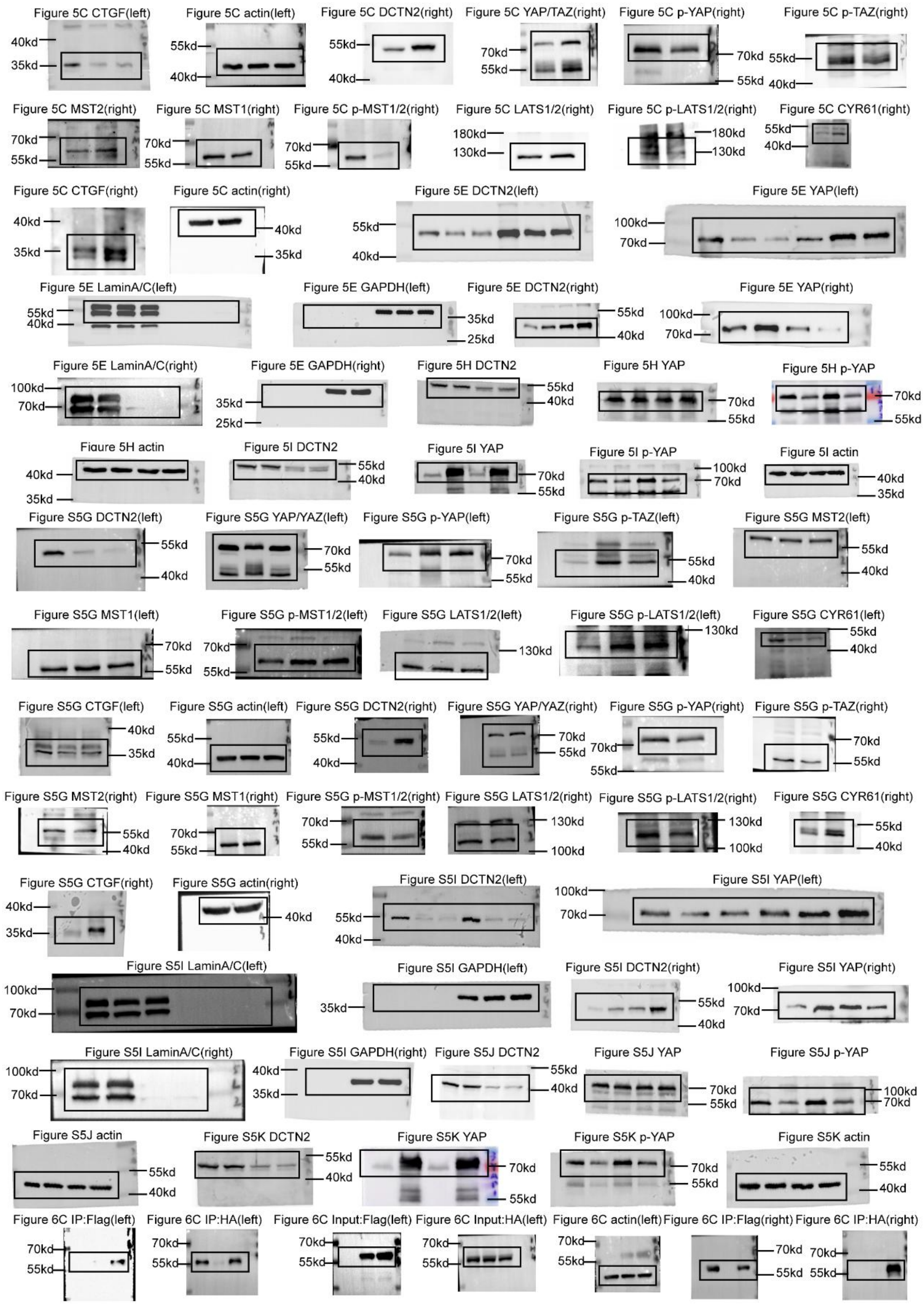

Figure 5C CTGF(left)
Figure 5C actin(left)
Figure 5C DCTN2(right)
Figure 5C YAP/TAZ(right)
Figure 5C p-YAP(right)
Figure 5C p-TAZ(right)
Figure 5C MST2(right)
Figure 5C MST1(right)
Figure 5C p-MST1/2(right)
Figure 5C LATS1/2(right)
Figure 5C p-LATS1/2(right)
Figure 5C CYR61(right)
Figure 5C CTGF(right)
Figure 5C actin(right)
Figure 5E DCTN2(left)
Figure 5E YAP(left)
Figure 5E LaminA/C(left)
Figure 5E GAPDH(left)
Figure 5E DCTN2(right)
Figure 5E YAP(right)
Figure 5E LaminA/C(right)
Figure 5E GAPDH(right)
Figure 5H DCTN2
Figure 5H YAP
Figure 5H p-YAP
Figure 5H actin
Figure 5I DCTN2
Figure 5I YAP
Figure 5I p-YAP
Figure 5I actin
Figure S5G DCTN2(left)
Figure S5G YAP/YAZ(left)
Figure S5G p-YAP(left)
Figure S5G p-TAZ(left)
Figure S5G MST2(left)
Figure S5G MST1(left)
Figure S5G p-MST1/2(left)
Figure S5G LATS1/2(left)
Figure S5G p-LATS1/2(left)
Figure S5G CYR61(left)
Figure S5G CTGF(left)
Figure S5G actin(left)
Figure S5G DCTN2(right)
Figure S5G YAP/YAZ(right)
Figure S5G p-YAP(right)
Figure S5G p-TAZ(right)
Figure S5G MST2(right)
Figure S5G MST1(right)
Figure S5G p-MST1/2(right)
Figure S5G LATS1/2(right)
Figure S5G p-LATS1/2(right)
Figure S5G CYR61(right)
Figure S5G CTGF(right)
Figure S5G actin(right)
Figure S5I DCTN2(left)
Figure S5I YAP(left)
Figure S5I LaminA/C(left)
Figure S5I GAPDH(left)
Figure S5I DCTN2(right)
Figure S5I YAP(right)
Figure S5I LaminA/C(right)
Figure S5I GAPDH(right)
Figure S5J DCTN2
Figure S5J YAP
Figure S5J p-YAP
Figure S5J actin
Figure S5K DCTN2
Figure S5K YAP
Figure S5K p-YAP
Figure S5K actin
Figure 6C IP:Flag(left)
Figure 6C IP:HA(left)
Figure 6C Input:Flag(left)
Figure 6C Input:HA(left)
Figure 6C actin(left)
Figure 6C IP:Flag(right)
Figure 6C IP:HA(right)

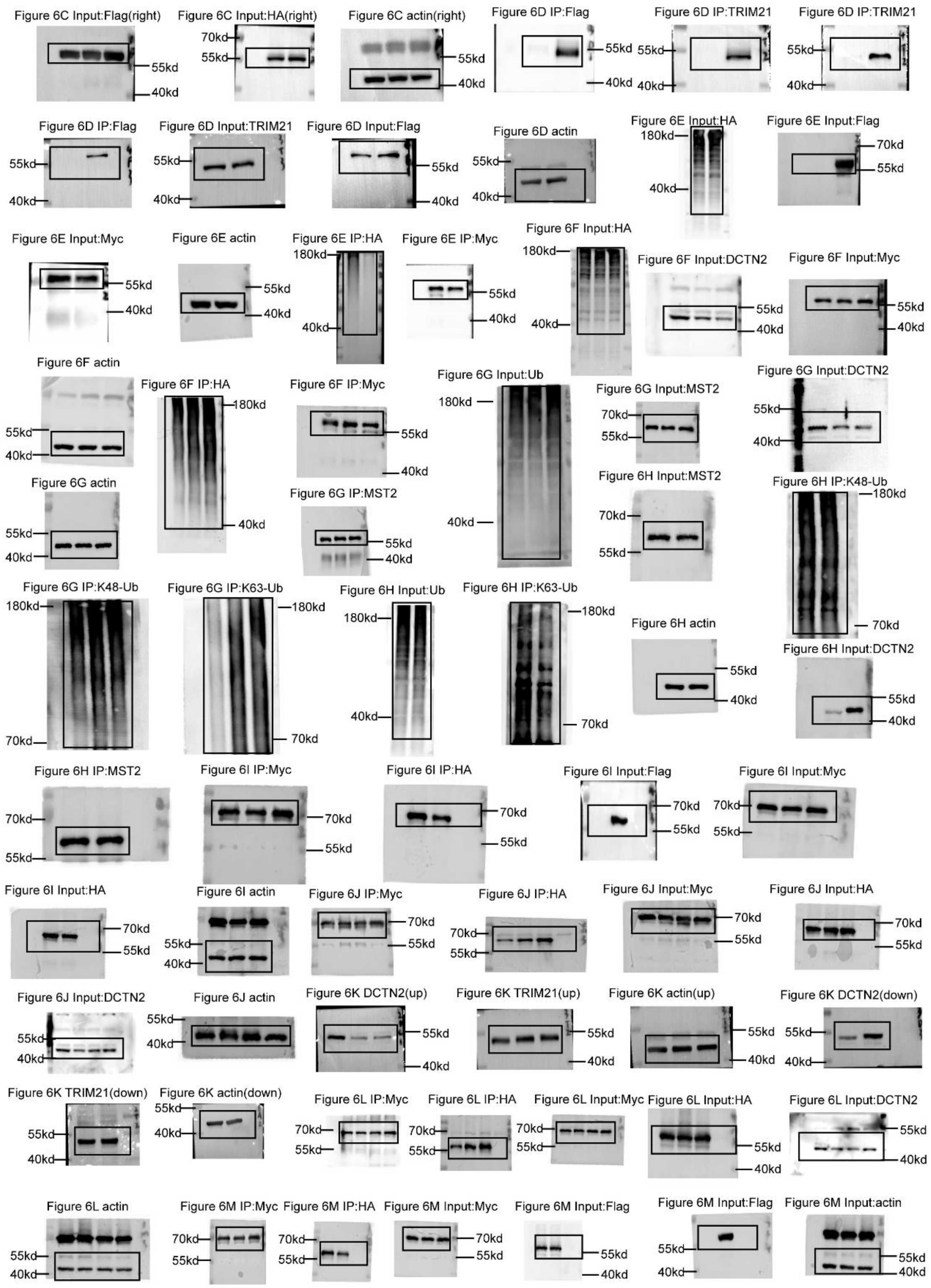

Figure 6C Input:Flag(right)
Figure 6C Input:HA(right)
Figure 6C actin(right)
Figure 6D IP:Flag
Figure 6D IP:TRIM21
Figure 6D IP:TRIM21
Figure 6D IP:Flag
Figure 6D Input:TRIM21
Figure 6D Input:Flag
Figure 6D actin
Figure 6E Input:HA
Figure 6E Input:Flag
Figure 6E Input:Myc
Figure 6E actin
Figure 6E IP:HA
Figure 6E IP:Myc
Figure 6F Input:HA
Figure 6F Input:DCTN2
Figure 6F Input:Myc
Figure 6F actin
Figure 6F IP:HA
Figure 6F IP:Myc
Figure 6G Input:Ub
Figure 6G Input:MST2
Figure 6G Input:DCTN2
Figure 6G actin
Figure 6G IP:MST2
Figure 6H Input:MST2
Figure 6H IP:K48-Ub
Figure 6G IP:K48-Ub
Figure 6G IP:K63-Ub
Figure 6H Input:Ub
Figure 6H IP:K63-Ub
Figure 6H actin
Figure 6H Input:DCTN2
Figure 6H IP:MST2
Figure 6I IP:Myc
Figure 6I IP:HA
Figure 6I Input:Flag
Figure 6I Input:Myc
Figure 6I Input:HA
Figure 6I actin
Figure 6J IP:Myc
Figure 6J IP:HA
Figure 6J Input:Myc
Figure 6J Input:HA
Figure 6J Input:DCTN2
Figure 6J actin
Figure 6K DCTN2(up)
Figure 6K TRIM21(up)
Figure 6K actin(up)
Figure 6K DCTN2(down)
Figure 6K TRIM21(down)
Figure 6K actin(down)
Figure 6L IP:Myc
Figure 6L IP:HA
Figure 6L Input:Myc
Figure 6L Input:HA
Figure 6L Input:DCTN2
Figure 6L actin
Figure 6M IP:Myc
Figure 6M IP:HA
Figure 6M Input:Myc
Figure 6M Input:Flag
Figure 6M Input:Flag
Figure 6M Input:actin

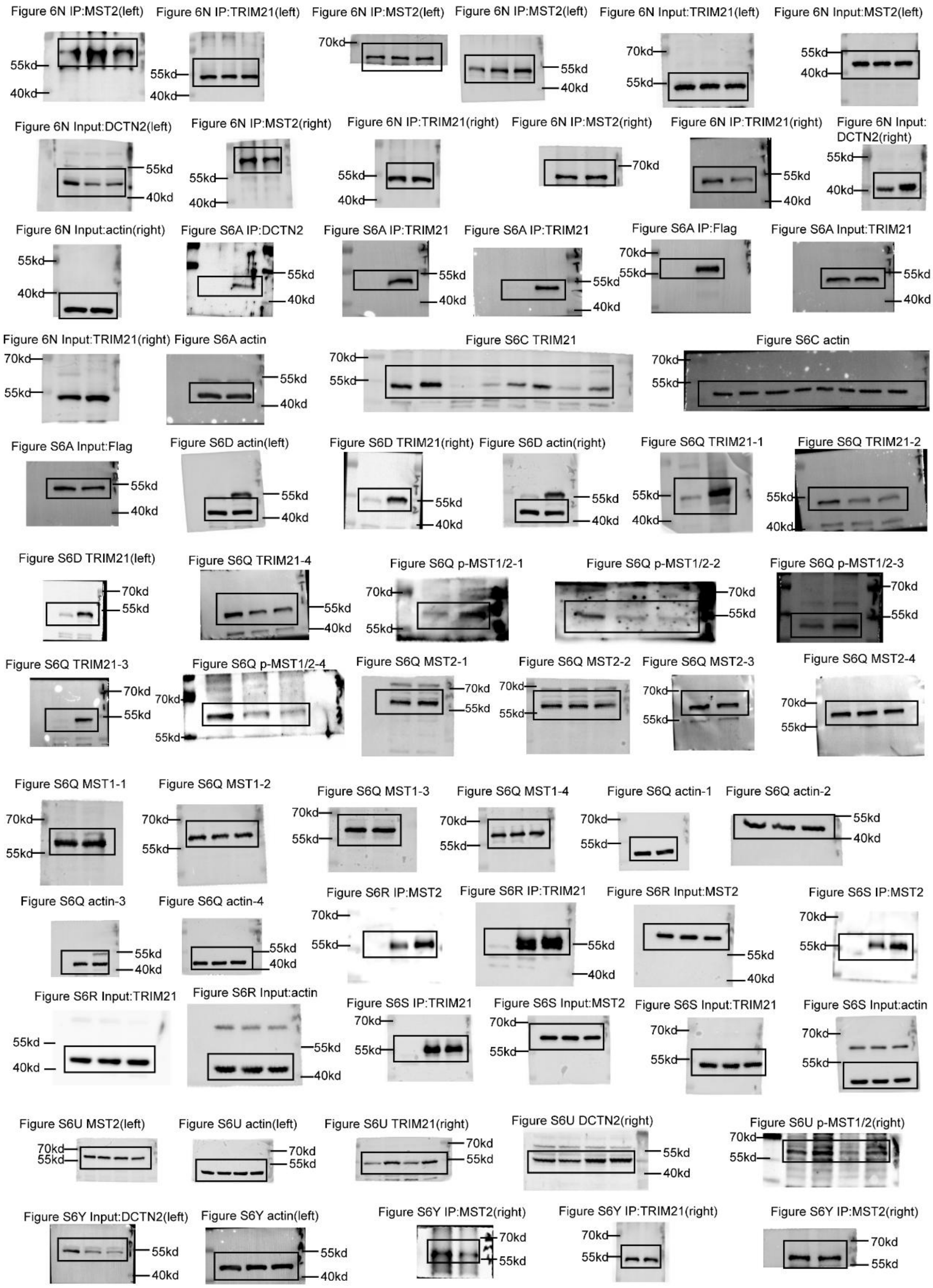
Figure 6N IP:MST2(left)
55kd 40kd
Figure 6N IP:TRIM21(left)
55kd 40kd
Figure 6N IP:MST2(left)
70kd
Figure 6N IP:MST2(left)
55kd 40kd
Figure 6N Input:TRIM21(left)
70kd 55kd
Figure 6N Input:MST2(left)
55kd 40kd
Figure 6N Input:DCTN2(left)
55kd 40kd
Figure 6N IP:MST2(right)
55kd 40kd
Figure 6N IP:TRIM21(right)
55kd 40kd
Figure 6N IP:MST2(right)
70kd
Figure 6N IP:TRIM21(right)
55kd 40kd
Figure 6N Input: DCTN2(right)
55kd 40kd
Figure 6N Input:actin(right)
55kd 40kd
Figure S6A IP:DCTN2
55kd 40kd
Figure S6A IP:TRIM21
55kd 40kd
Figure S6A IP:TRIM21
55kd 40kd
Figure S6A IP:Flag
70kd 55kd
Figure S6A Input:TRIM21
55kd 40kd
Figure 6N Input:TRIM21(right)
70kd 55kd
Figure S6A actin
55kd 40kd
Figure S6C TRIM21
70kd 55kd
Figure S6C actin
70kd 55kd
Figure S6A Input:Flag
55kd 40kd
Figure S6D actin(left)
55kd 40kd
Figure S6D TRIM21(right)
55kd 40kd
Figure S6D actin(right)
55kd 40kd
Figure S6Q TRIM21-1
55kd 40kd
Figure S6Q TRIM21-2
55kd 40kd
Figure S6D TRIM21(left)
70kd 55kd
Figure S6Q TRIM21-4
55kd 40kd
Figure S6Q p-MST1/2-1
70kd 55kd
Figure S6Q p-MST1/2-2
70kd 55kd
Figure S6Q p-MST1/2-3
70kd 55kd
Figure S6Q TRIM21-3
70kd 55kd
Figure S6Q p-MST1/2-4
70kd 55kd
Figure S6Q MST2-1
70kd 55kd
Figure S6Q MST2-2
70kd 55kd
Figure S6Q MST2-3
70kd 55kd
Figure S6Q MST2-4
70kd 55kd
Figure S6Q MST1-1
70kd 55kd
Figure S6Q MST1-2
70kd 55kd
Figure S6Q MST1-3
70kd 55kd
Figure S6Q MST1-4
70kd 55kd
Figure S6Q actin-1
70kd 55kd
Figure S6Q actin-2
55kd 40kd
Figure S6Q actin-3
55kd 40kd
Figure S6Q actin-4
55kd 40kd
Figure S6R IP:MST2
70kd 55kd
Figure S6R IP:TRIM21
55kd 40kd
Figure S6R Input:MST2
55kd 40kd
Figure S6S IP:MST2
70kd 55kd
Figure S6R Input:TRIM21
55kd 40kd
Figure S6R Input:actin
55kd 40kd
Figure S6S IP:TRIM21
70kd 55kd
Figure S6S Input:MST2
70kd 55kd
Figure S6S Input:TRIM21
70kd 55kd
Figure S6S Input:actin
70kd 55kd
Figure S6U MST2(left)
70kd 55kd
Figure S6U actin(left)
70kd 55kd
Figure S6U TRIM21(right)
70kd 55kd
Figure S6U DCTN2(right)
55kd 40kd
Figure S6U p-MST1/2(right)
70kd 55kd
Figure S6Y Input:DCTN2(left)
55kd 40kd
Figure S6Y actin(left)
55kd 40kd
Figure S6Y IP:MST2(right)
70kd 55kd
Figure S6Y IP:TRIM21(right)
70kd 55kd
Figure S6Y IP:MST2(right)
70kd 55kd

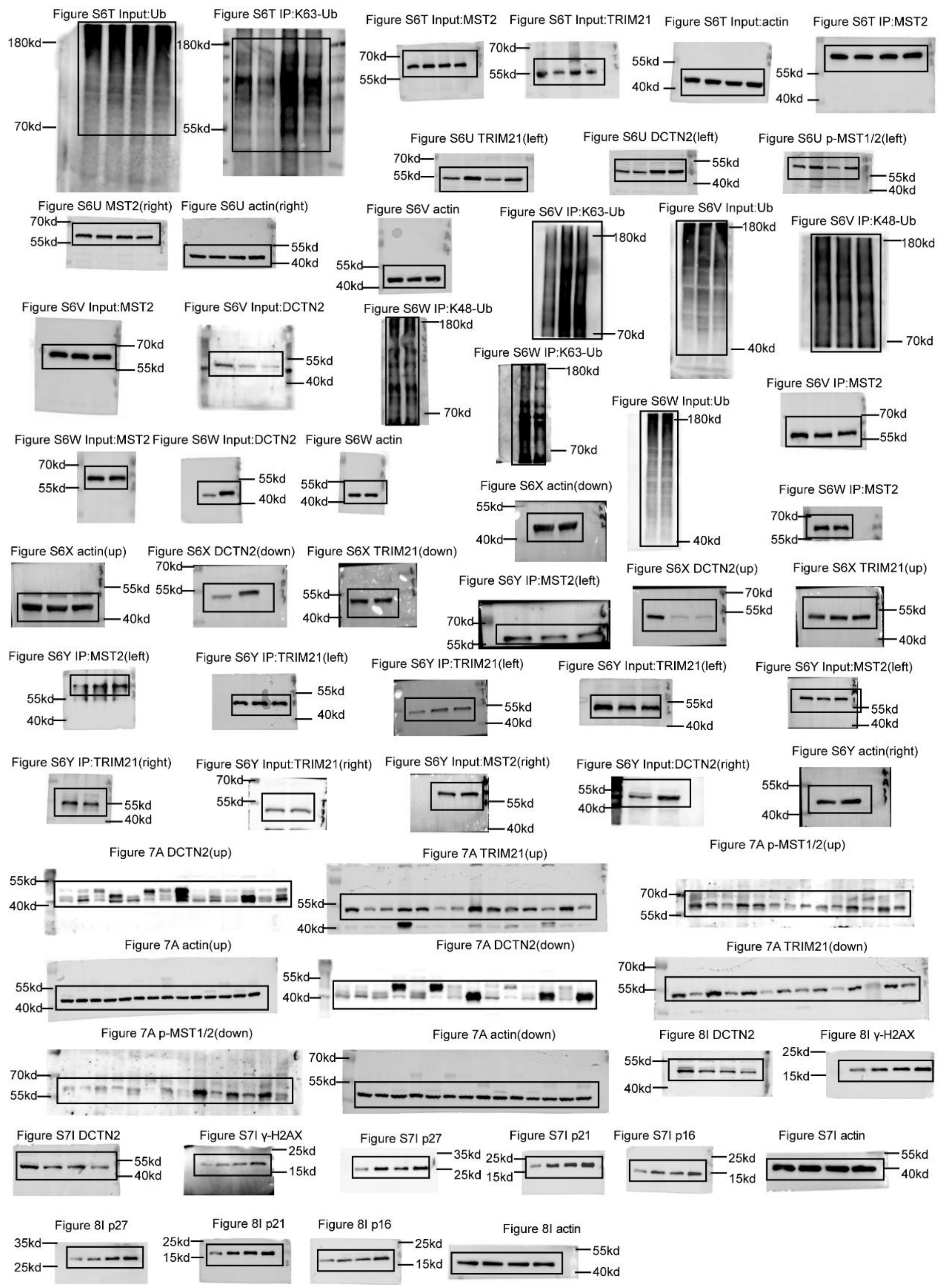
Figure S6T Input:Ub
180kd
70kd
Figure S6T IP:K63-Ub
180kd
55kd
Figure S6T Input:MST2
70kd
55kd
Figure S6T Input:TRIM21
70kd
55kd
Figure S6T Input:actin
55kd
40kd
Figure S6T IP:MST2
55kd
40kd
Figure S6U TRIM21(left)
70kd
55kd
Figure S6U DCTN2(left)
55kd
40kd
Figure S6U p-MST1/2(left)
55kd
40kd
Figure S6U MST2(right)
70kd
55kd
Figure S6U actin(right)
55kd
40kd
Figure S6V actin
55kd
40kd
Figure S6V IP:K63-Ub
180kd
70kd
Figure S6V Input:Ub
180kd
40kd
Figure S6V IP:K48-Ub
180kd
70kd
Figure S6V Input:MST2
70kd
55kd
Figure S6V Input:DCTN2
55kd
40kd
Figure S6W IP:K48-Ub
180kd
70kd
Figure S6W IP:K63-Ub
180kd
70kd
Figure S6W Input:Ub
180kd
40kd
Figure S6V IP:MST2
70kd
55kd
Figure S6W Input:MST2
70kd
55kd
Figure S6W Input:DCTN2
55kd
40kd
Figure S6W actin
55kd
40kd
Figure S6X actin(down)
55kd
40kd
Figure S6W IP:MST2
70kd
55kd
Figure S6X actin(up)
55kd
40kd
Figure S6X DCTN2(down)
70kd
55kd
Figure S6X TRIM21(down)
55kd
40kd
Figure S6Y IP:MST2(left)
70kd
55kd
Figure S6X DCTN2(up)
70kd
55kd
Figure S6X TRIM21(up)
55kd
40kd
Figure S6Y IP:MST2(left)
55kd
40kd
Figure S6Y IP:TRIM21(left)
55kd
40kd
Figure S6Y IP:TRIM21(left)
55kd
40kd
Figure S6Y Input:TRIM21(left)
55kd
40kd
Figure S6Y Input:MST2(left)
55kd
40kd
Figure S6Y IP:TRIM21(right)
55kd
40kd
Figure S6Y Input:TRIM21(right)
70kd
55kd
Figure S6Y Input:MST2(right)
55kd
40kd
Figure S6Y Input:DCTN2(right)
55kd
40kd
Figure S6Y actin(right)
55kd
40kd
Figure 7A DCTN2(up)
55kd
40kd
Figure 7A TRIM21(up)
55kd
40kd
Figure 7A p-MST1/2(up)
70kd
55kd
Figure 7A actin(up)
55kd
40kd
Figure 7A DCTN2(down)
55kd
40kd
Figure 7A TRIM21(down)
70kd
55kd
Figure 7A p-MST1/2(down)
70kd
55kd
Figure 7A actin(down)
70kd
55kd
Figure 8I DCTN2
55kd
40kd
Figure 8I γ-H2AX
25kd
15kd
Figure S7I DCTN2
55kd
40kd
Figure S7I γ-H2AX
25kd
15kd
Figure S7I p27
35kd
25kd
Figure S7I p21
25kd
15kd
Figure S7I p16
25kd
15kd
Figure S7I actin
55kd
40kd
Figure 8I p27
35kd
25kd
Figure 8I p21
25kd
15kd
Figure 8I p16
25kd
15kd
Figure 8I actin
55kd
40kd
